# Genomics reveal population structure, evolutionary history, and signatures of selection in the northern bottlenose whale, *Hyperoodon ampullatus*

**DOI:** 10.1111/mec.16643

**Published:** 2022-08-23

**Authors:** Evelien de Greef, Anthony L. Einfeldt, Patrick J. O. Miller, Steven H. Ferguson, Colin J. Garroway, Kyle J. Lefort, Ian G. Paterson, Paul Bentzen, Laura J. Feyrer

**Affiliations:** ^1^ Department of Biology Dalhousie University Halifax Nova Scotia Canada; ^2^ Department of Biological Sciences University of Manitoba Winnipeg Manitoba Canada; ^3^ School of Biology University of St. Andrews St. Andrews UK; ^4^ Fisheries and Oceans Canada Freshwater Institute Winnipeg Manitoba Canada; ^5^ Fisheries and Oceans Canada Bedford Institute of Oceanography Dartmouth Nova Scotia Canada

**Keywords:** cetacean, conservation, genetic diversity, genomics, whale

## Abstract

Information on wildlife population structure, demographic history, and adaptations are fundamental to understanding species evolution and informing conservation strategies. To study this ecological context for a cetacean of conservation concern, we conducted the first genomic assessment of the northern bottlenose whale, *Hyperoodon ampullatus*, using whole‐genome resequencing data (*n* = 37) from five regions across the North Atlantic Ocean. We found a range‐wide pattern of isolation‐by‐distance with a genetic subdivision distinguishing three subgroups: the Scotian Shelf, western North Atlantic, and Jan Mayen regions. Signals of elevated levels of inbreeding in the Endangered Scotian Shelf population indicate this population may be more vulnerable than the other two subgroups. In addition to signatures of inbreeding, evidence of local adaptation in the Scotian Shelf was detected across the genome. We found a long‐term decline in effective population size for the species, which poses risks to their genetic diversity and may be exacerbated by the isolating effects of population subdivision. Protecting important habitat and migratory corridors should be prioritized to rebuild population sizes that were diminished by commercial whaling, strengthen gene flow, and ensure animals can move across regions in response to environmental changes.

## 1 INTRODUCTION

The health of wildlife populations and their resilience to changing environments is influenced by many intrinsic and extrinsic dynamics that can lead to an increased risk of extinction. Declining genetic diversity in a population is one factor that can lead to detrimental outcomes, including inbreeding depression and loss of adaptive evolutionary potential. Understanding the processes that underlie contemporary distributions of genetic variation can guide conservation strategies to preserve and recover at‐risk populations. Genomic approaches are becoming increasingly important for delineating population subdivisions and investigating genetic variation to inform regional management targets for conservation priority (Funk et al., 2012). For example, Reeves et al. (2022) examined killer whales (*Orcinus orca*) and distinguished three main genetic populations in the Australasian region, providing a framework for management designations.

Genetic information can also be used to reconstruct historical patterns of population size, using the timing of patterns to infer whether changes are associated with long‐term natural processes or shorter‐term human activities. Climatic oscillations in the Pleistocene affected distributions and abundances of marine species through changes in environmental factors such as ocean temperature and sea level fluctuations (Maggs et al., 2008). Some species experienced declines in effective population size (*N*_e_) during these periods, as observed in the widespread population bottleneck in killer whales (Moura et al., 2014). However, demographic histories have varied among cetacean species (aquatic mammals including whales, dolphins, and porpoises). For example, some deep‐diving cetaceans maintained a relatively stable *N*_e_ over the past million years, seen in the Grey's beaked whale, *Mesoplodon grayi* (Westbury et al., 2021), or exhibited population fluctuations, observed in the sperm whale, *Physeter macrocephalus* (Morin et al., 2018), followed by a recent increase in parallel with potential expansion of suitable habitat. In contrast, the *N*_e_ of narwhals, *Monodon monoceros* (Westbury, Petersen, Garde, et al., 2019) and Ramari's beaked whale, *Mesoplodon eueu* (Carroll et al., 2021) gradually declined and then expanded, coinciding with the onset of the last glacial period. Long‐term declines in genetic diversity may leave species vulnerable to contemporary and emerging anthropogenic threats, such as vessel strikes and entanglement (e.g., Moore et al., 2021), and predicted prey reduction due to climate warming (e.g., Tulloch et al., 2019). Such threats can accelerate negative population‐level effects and increase the risk of inbreeding depression.

Genomic studies using whole‐genome sequencing (e.g., Wheeler et al., 2008) and reduced‐representation sequencing (e.g., Luca et al., 2011) examine genetic material across the entire genome of an organism, which is particularly important for understanding population structure and targets of selection for species with low genetic diversity. Compared to other mammalian taxa, cetaceans generally have low genetic diversity, which is thought to be influenced by their long lifespans and generation times (Vachon et al., 2018), and slower mutation rates (Tollis et al., 2019). The northern bottlenose whale, *Hyperoodon ampullatus*, has one of the lowest known genetic diversities among all cetaceans (Feyrer et al., 2019; Louis et al., 2020). While traditional genetic approaches for studying population genetics (e.g., using mitochondrial DNA [mtDNA] and microsatellite data; Baker et al., 1994; Feyrer et al., 2019; Morin et al., 2010) can provide valuable insights, they only investigate a small number of targeted genetic markers or those with matrilineal inheritance. Patterns detected using traditional methods may not be representative of the entire genome, potentially leading to biased inferences about a species' diversity and demographic history (Nabholz et al., 2008). As the accessibility of whole‐genome sequencing has rapidly increased (Cammen et al., 2016), the improved resolution and power of these tools allow researchers to explore the assumptions and patterns previously identified with traditional genetic markers. A growing number of studies have demonstrated the power of genomics to detect subtle patterns of population structure and adaptive differentiation, expanding our understanding of evolutionary processes in a range of aquatic taxa including cetaceans (Foote et al., 2016; Warren et al., 2017; Westbury et al., 2021) to turtles (Gallego‐García et al., 2021), and fishes (Petrou et al., 2021).

The northern bottlenose whale is a species of beaked whale endemic to the deep waters (>500 m) of the North Atlantic Ocean, primarily found along the edge of the continental slope (Whitehead & Hooker, 2012). Their foraging dives regularly reach depths over 800 m (Hooker & Baird, 1999), with the deepest dive recorded at 2339 m (Miller et al., 2015), making the northern bottlenose whale one of the deepest‐diving mammals. The species' southern‐most population, which inhabits the Scotian Shelf, is listed as Endangered under Canada's Species At Risk Act (COSEWIC, 2011). Individuals in this region have been observed to exhibit high site fidelity (Feyrer et al., 2021). Previous work using mitogenomes and microsatellites has identified this population as genetically differentiated from the northern regions (Einfeldt et al., 2022; Feyrer et al., 2019). Despite being one of the few beaked whale species subject to long‐term studies (Hooker et al., 2019), their conservation status remains uncertain following large‐scale commercial whaling in the 19th–20th centuries and other anthropogenic threats such as entanglement across their range (Feyrer et al., 2021; Whitehead & Hooker, 2012). Whaling could have reduced genetic diversity (Alter et al., 2012), both directly through removal and indirectly via increased effects of genetic drift such as inbreeding depression. Previous work on northern bottlenose whale mitogenomes identified a sharp demographic decline in the Scotian Shelf population consistent with human activity (Feyrer et al., 2019). However, because mitogenomes are a single linked matrilineal marker it is possible that the observed changes in *N*_e_ do not reflect species‐wide changes in demography or may reflect other processes such as selective sweeps (Morin et al., 2018), and it is unclear whether observed declines in *N*_e_ were due to either natural processes or whaling. Given estimates of restricted dispersal among regions, distinctions between the Scotian Shelf population and the northern regions (Feyrer et al., 2019) also bring to question the role of adaptation to local selection pressures. Geographic variation between subgroups spans latitudinal gradients and reflects unique regional conditions, where differences in climatic factors (e.g., temperature) or ecosystem structure could contribute to different evolutionary trajectories. Studying genomic data available for northern bottlenose whales, is also relevant to understanding the evolutionary and ecological mechanisms distinguishing populations of other cetaceans and marine mammals, and can help guide future research and conservation decisions (Gallego‐García et al., 2021).

To address these questions and expand our understanding of genetic patterns in the northern bottlenose whale, we integrated a draft reference genome with whole‐genome resequencing data from five regions representing the known distribution across the North Atlantic Ocean: the Scotian Shelf, Newfoundland, Southern Labrador, the Canadian Arctic (Davis Strait), and Jan Mayen (Iceland). Our objective was to use high‐resolution genomic data to examine northern bottlenose whale population structure, demographic history, changes in genetic diversity, and for the first time, to consider indices of inbreeding and evidence for selection across geographic regions in the North Atlantic.

## 2 MATERIALS AND METHODS

### 2.1 Reference genome

The northern bottlenose whale reference genome was derived from an individual in the Scotian Shelf. The tissue sample was collected by dart biopsy from a live animal in 2016 as described in Feyrer et al. (2019). DNA was extracted using a phenol‐chloroform protocol (Sambrook & Russell, 2006). Data were sequenced in collaboration with CanSeq150 using 10×‐genomics linked reads, and then assembled using Supernova (Weisenfeld et al., 2017). The reference genome was assessed with Assemblathon2 (Bradnam et al., 2013) and completeness was evaluated by comparison to conserved mammalian orthologues with BUSCO version 4.1.4 (Seppey et al., 2019). Cetaceans have broad conservation of chromosomal arrangement (Yuan et al., 2021), thus using a chromosome‐level genome of a different species can help determine scaffold alignment with mammalian chromosomes (e.g., genomic regions making up chromosome 1). We mapped the reference genome to the annotated blue whale (*Balaenoptera musculus*) reference genome obtained from NCBI database (accession no. GCA_009873245.3) with SatsumaSynteny version 2 (Grabherr et al., 2010). We annotated the reference genome to identify and locate genes using MAKER version 2.31.10 (Holt & Yandell, 2011) by generating gene models using protein data obtained from *Ensembl* database (Cunningham et al., 2019) for three model species: blue whale, sperm whale (*Physeter macrocephalus*), and cow (*Bos taurus*).

### 2.2 Resequencing data

**FIGURE 1:**
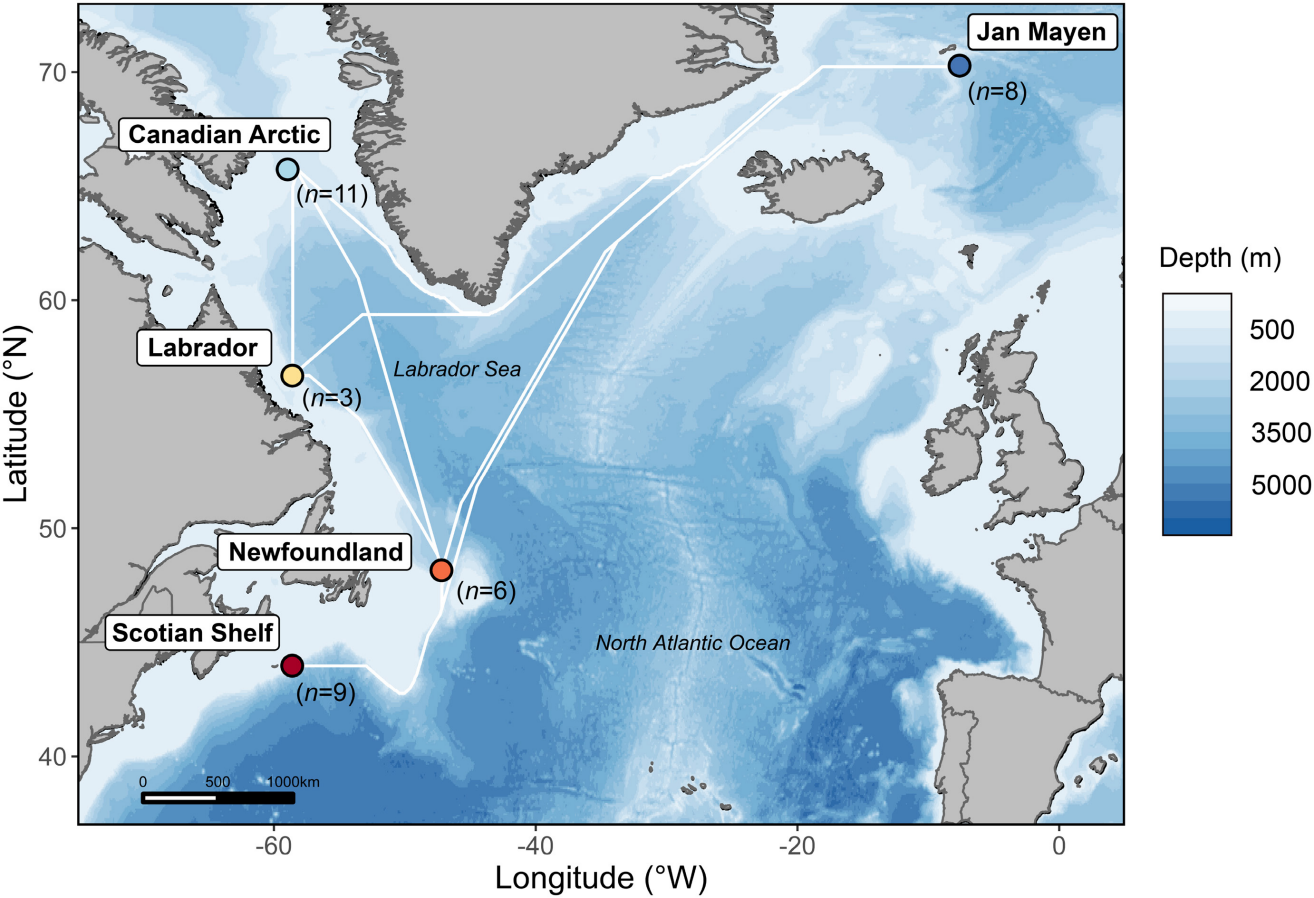
Map of northern bottlenose whale sample sites across the North Atlantic Ocean, collected between 2003–2019. Dark blue, Jan Mayen; light blue, Canadian Arctic; yellow, Labrador; orange, Newfoundland; red, Scotian shelf. White lines represent shortest distances between sites in water depths of 500 m or greater.

Forty‐nine tissue samples for resequencing data were collected from live wild animals (2003–2019) in the North Atlantic Ocean, specifically in the Scotian Shelf, Newfoundland, Labrador, Canadian Arctic, and Jan Mayen regions (Figure 1). DNA was extracted using the same methodology as the reference genome sample and libraries were prepared with Illumina's Nextera XT DNA Library Preparation Kit and Illumina's Nextera DNA Flex Library Prep Kit. The samples were sequenced on an Illumina NovaSeq platform to produce whole‐genome resequencing data. Raw sequencing files were trimmed with Trimmomatic version 0.36 (Bolger et al., 2014). We mapped the reads to the northern bottlenose whale reference genome with BWA version 0.7.17 (Li & Durbin, 2009), then sorted and indexed with SAMtools version 1.9 (Li et al., 2009). We removed duplicate reads and added read group information with Picard version 2.20.6 (Broad Institute, 2019). To account for coverage variation across samples (range 1–11×) that can influence variant calling, we adjusted sequencing depth for 22 samples using GATK version 4.1.2 (McKenna et al., 2010) to 5× modal coverage. Using the draft reference genome as a backbone, we called genomic variants with the sequencing data for all samples using Platypus version 0.8.1 (Rimmer et al., 2014). We used VCFtools version 0.1.17 (Danecek et al., 2011) and GATK version 4.1.2 (McKenna et al., 2010) to filter single‐nucleotide polymorphisms (SNPs), removing insertions and deletions (indels), low‐quality sites (QUAL < 20, MQ < 30, QD <2), SNPs with more than 40% missing data and nonbiallelic sites. We identified sex‐linked scaffolds through coverage comparisons between male and female samples (Grayson et al., 2022) with DifCover (Smith et al., 2018), then filtered SNPs from these regions to create an autosomal data set (see Appendix S1 for identifying X and Y chromosomes). Due to the potential effects of structural variants on population analyses (Seich al Basatena et al., 2013), we used BreakDancer version 1.3.6 (Fan et al., 2014) to identify putative inversions and translocations, which were filtered out from downstream analyses. Individuals with over 40% missing data were removed from the data set. Given that relatedness, or kinship, may bias population analyses (Louis et al., 2014; O'Connell et al., 2019), we also identified kin pairs by estimating identity‐by‐descent with PLINK version 1.9 (Purcell et al., 2007), using the pi‐hat value of 0.4 as a threshold to remove first‐degree relatives. From each pair identified, we removed the sample with the higher amount of missing data.

### 2.3 Population structure

After further filtering the SNP data set for minor allele count of two, removing scaffolds less than 50 kb in length, removing sites out of Hardy–Weinberg equilibrium (HWE) (using an observed heterozygous frequency threshold of 0.6 across all sampling locations), and pruning for linkage‐disequilibrium (LD *r* ^2^ > 0.5) with VCFtools version 0.1.17 (Danecek et al., 2011) and PLINK version 1.9 (Purcell et al., 2007), we examined interindividual genomic variation using principal component analyses (PCA) with pcadapt version 4.3.3 (Privé et al., 2020). Ancestral admixture analyses were executed using sparse non‐negative matrix factorization (sNMF) in LEA version 3.3.2 (Frichot & François, 2015) using values of K from 1 to 5. Given the PCA‐based approach and imputation of missing data in sNMF, which was performed through resampling missing genotypes and updating missing values with predictive probabilities (Frichot et al., 2014), we filtered out SNPs with more than 10% missing data to minimize potential biases from imputation for this specific admixture analysis. Estimates of genetic diversity were measured with expected and observed heterozygosity using hierfstat version 0.5–7 (Goudet, 2004). We calculated pairwise estimates of *F*_ST_ using an estimator introduced by Reich et al. (2009) to avoid biases from small sample sizes, then adjusted values to Slatkin's *F*_ST_ (Slatkin, 1995). We measured distances between sites with marmap (Pante & Simon‐Bouhet, 2013) within ocean depths minimum of 500 m (to account for connectivity between areas of deep water habitat) and evaluated isolation‐by‐distance (IBD) through correlations of *F*_ST_ and site distances with a Mantel test using 9999 permutations in ade4 version 1.7–16 (Dray & Dufour, 2007). Additionally, we assessed correlations of *F*_ST_ with latitude between sites as an initial exploration of correlates corresponding with environmental differentiation.

### 2.4 Demographic history

We estimated changes in *N*_e_ through coalescent‐based inferences using *SMC++*, which combines site frequency spectrum and linkage information from multiple unphased samples (Terhorst et al., 2017). Because SMC models are influenced by genome continuity, we removed SNPs aligned to scaffolds below 100 kb in length (Gower et al., 2018), and additionally removed SNPs out of HWE (using an observed heterozygous frequency threshold of 0.6 across all sampling locations) with VCFtools version 0.1.17 (Danecek et al., 2011). Indels and unused loci were provided as a masked file create through bedops (Neph et al., 2012). We used a generation time of 17.8 years (Taylor et al., 2007) and a mutation rate of 1.53 × 10^−8^ substitution/nucleotide/generation, representing an average of published mutation rates for cetaceans (Moura et al., 2014). To prepare distinguished lineages, we used an individual from each sampling site to form the distinguished pair in *SMC++*, and ran 20 iterations with each one separately, totalling 100 iterations with samples grouped as one population. Due to evidence of geographical differentiation, we also estimated demographic history within three subgroups: Jan Mayen, western North Atlantic (Canadian Arctic, Labrador, Newfoundland), and the Scotian Shelf.

### 2.5 Inbreeding

To evaluate levels of inbreeding, we calculated runs of homozygosity (ROH). In a population with higher levels of inbreeding, a greater number and lengths of ROHs are expected (Curik et al., 2014). Using the same SNP data set from the previous population structure analyses, we estimated ROHs across individual genomes (Foote et al., 2021) for each genetic subgroup (Jan Mayen, western North Atlantic, and the Scotian Shelf), through PLINK version 1.9 (Purcell et al., 2007) with a minimum segment length of 50 kb and a minimum number of 50 SNPs, selecting the default parameters for sliding windows (50 SNPs, max 1 heterozygous call and 5 missing calls, and a hit rate threshold of 0.05).

### 2.6 Regions under selection

A rapid rise in the frequency of beneficial alleles (a selective sweep) is accompanied by a reduction in haplotype diversity due to a “hitch‐hiking” effect, allowing genomic regions under selection to be located from haplotype structure (Stephan, 2019). Extended haplotype homozygosity (EHH) measures reduced haplotype diversity and is reliable for detecting regions under recent selective pressure (Bomba et al., 2015). As opposed to examining genomic differentiation (*F*_ST_), dependent on levels of diversity (Cruickshank & Hahn, 2014), we measured EHH to examine signatures of selection where we expected subtle differences between geographic regions including scenarios where there is polymorphism in regions where selection pressures are absent. We used a cross‐population test (XP‐EHH) between the Scotian Shelf and all other regions (Jan Mayen, western North Atlantic) to estimate alleles that have risen to near fixation in one population (Sabeti et al., 2007) using the program rehh (Gautier & Vitalis, 2012). We additionally completed XP‐EHH analyses with Jan Mayen and western North Atlantic groups in separate comparisons with the Scotian Shelf. SNPs were further filtered to a max‐missingness of 10% and minor allele count of two. We removed scaffolds less than 50 kb in length and then imputed and phased SNPs with beagle version 5.2 (Browning et al., 2018, 2021), a method using identity‐by‐descent segments and hidden Markov models. We used default parameters for imputation (1600 model states, 6 cM of haplotype segments, 0.1 cM step size, and 7 consecutive steps), and 20 iterations to estimate genotype phase (with a maximum of 3 burnin iterations and 280 model states). We used the ies2xpehh function in rehh to calculate the pairwise XP‐EHH statistics. To determine candidate regions, we used the calc_candidate_regions function and set a threshold of ‐log_10_(0.05), representing a 0.05 *p*‐value cutoff, and a window size of 100 kb overlapping by 10 kb with a minimum of two significant markers. Genes located within 20 kb of the significant SNPs were pulled from the reference genome annotation using MAGMA (de Leeuw et al., 2015), and then analysed within each population with Enrichr (Chen et al., 2013) for gene ontology (GO) enrichment to identify groups of genes with shared functional characteristics.

## 3 RESULTS

### 3.1 Reference genome

The northern bottlenose whale reference genome assembly was 2.3 Gb in length, consisting of 67,191 scaffolds. BUSCO analyses detected 76% complete mammalian orthologues (Table S1). Chromosomal proportions from the synteny alignment ranged between 91%–98% of each blue whale autosome, and scaffolds not confidently matched to a chromosome were classified as “unplaced” and contained a total of 6% of the northern bottlenose whale genome (Figure S1). The genome annotation resulted in 53,629 genes, which is probably inflated due to the high number of scaffolds in the reference genome causing genes to be split across multiple scaffolds. The number of unique gene identifications was 15,223, which is consistent with the genome BUSCO score of 76% and an expected number of approximately 20,000 genes in a whale genome (Westbury, Petersen, & Lorenzen, 2019).

### 3.2 Resequencing data

The genetic variant data set for all 49 individuals (average 3.7× modal coverage) resulted in 7,636,698 variants, which were filtered down to 3,891,367 SNPs before applying further analysis‐specific filters such as minor allele frequency. Sample coverages are shown in Table S2, filtering steps are displayed in Figure S2, and distributions of SNP metrics for population structure analysis are shown in Figure S3. Coverage comparisons between male and female sequencing data (Figure S4) identified 4701 X‐linked scaffolds (103.6 Mb) and 984 Y‐linked scaffolds (7.1 Mb). Seven individuals were removed from further analysis due to high missingness. We also identified and removed one individual from each of four duplicate pairs and one kin pair. Our final sample size was 37 (average 4.1× modal coverage) (Table 1).

**TABLE 1:** Site locations for northern bottlenose whale samples (*N* = 37) across five regions, with mean location coordinates, region size (represented by the maximum distance between sampling locations), number of samples (including male and female ratio in parentheses), years and months of sample collection, expected heterozygosity (*H*_e_), and observed heterozygosity (*H*_o_). 995,508 SNPs were used in *H*_e_ and *H*_o_ analyses

Region	Lat.	Long.	Size (km)	*n* (M:F)	Year(s)	Month(s)	*H*_e_	*H*_o_
Jan Mayen	70.273	−7.610	249	8 (4:4)	2014	June	0.176	0.179
Canadian Arctic	65.727	−58.956	499	11 (9:2)	2018–2019	July–October	0.176	0.177
Labrador	56.686	−58.594	150	3 (2:1)	2003	August	0.170	0.172
Newfoundland	48.155	−47.197	27	6 (4:2)	2016–2017	June–July	0.177	0.184
Scotian Shelf	43.975	–8.602	88	9 (2:7)	2016–2019	July–August	0.174	0.178

### 3.3 Population structure analyses

**FIGURE 2:**
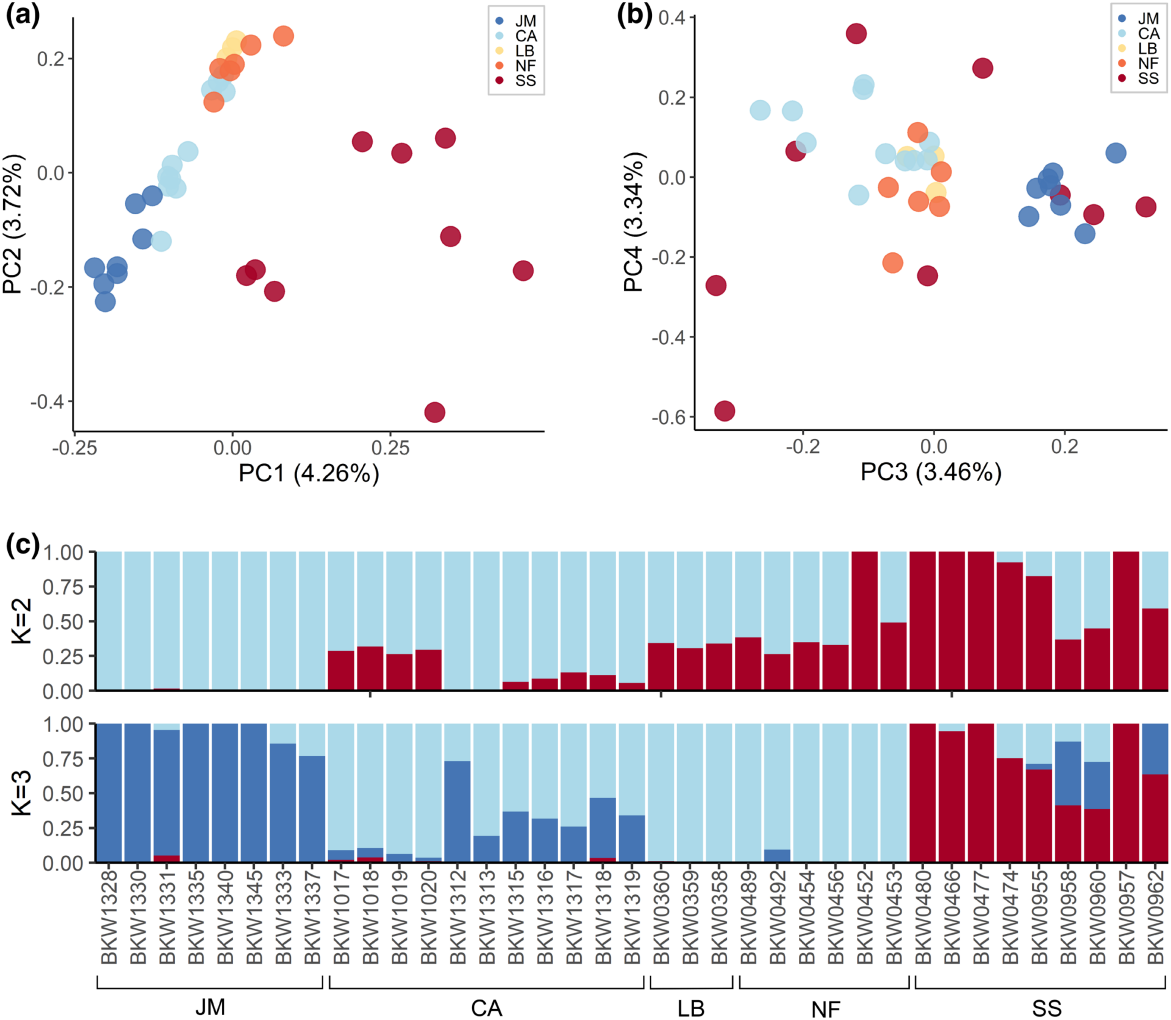
Regional clustering seen from northern bottlenose whale population analyses. (a) Principal components analysis (PCA) with first two principal axes and (b) PCA with third and fourth principal axes, where proportion of variance explained by each principal component is listed in parentheses, and (c) admixture results from sNMF analyses using *K* = 2 and *K* = 3 clusters. JM, Jan Mayen; CA, Canadian Arctic; LB, Labrador; NF, Newfoundland; SS, Scotian shelf. 995,508 SNPs were used in PCA, and 544,733 SNPs were used in sNMF analyses.

**FIGURE 3:**
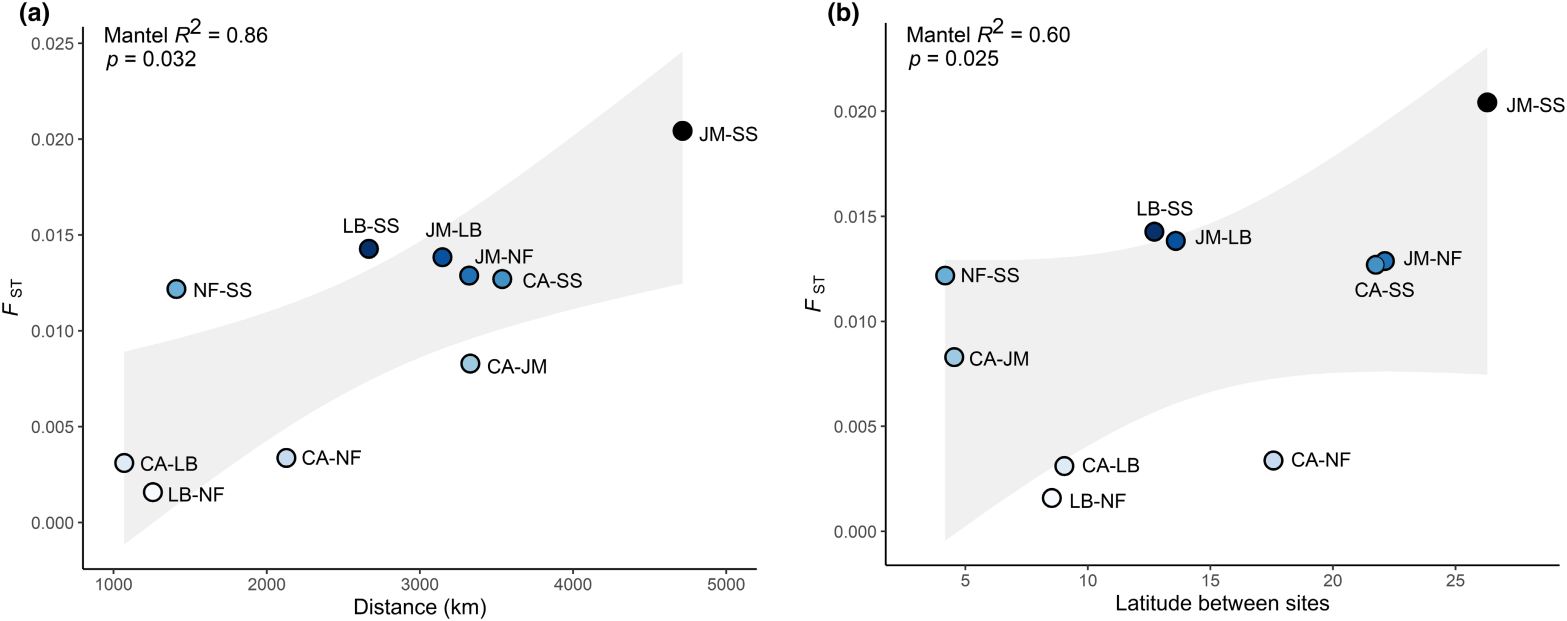
Correlations of pairwise *F*_ST_ from 995,508 SNPs with (a) distance and (b) latitude between northern bottlenose whale sampling locations (JM, Jan Mayen; CA, Canadian Arctic; LB, Labrador; NF, Newfoundland; SS, Scotian shelf). Linear regression standard error is coloured in grey. Mantel test correlation and *p*‐value are displayed top left.

The LD‐pruned data set resulted in 995,508 SNPs. The first two principal axes corresponded to latitude and distinguished individuals in the Scotian Shelf region as a separate group respectively (Figure 2a; see Figure S5 for PCA on sex‐linked SNPs). The third principal axis (Figure 2b) differentiated individuals in Jan Mayen from those in the Canadian Arctic, Labrador, and Newfoundland. Although individuals from the latter three areas show some overlap in the top three PCs, the distribution of clustering suggests there may be subtle structure between the Canadian Arctic and Newfoundland. Admixture results from sNMF (544,733 SNPs) were consistent with population structure observed in PCA, when *K* = 2 genetic clusters supported the Scotian Shelf as a separate ancestral genetic source, and *K* = 3 additionally supported Jan Mayen as a genetic source (Figure 2c). Cross‐entropy (CE) score results were similar across values of K under 3 and we selected models using K = 2 (CE = 0.53) and *K* = 3 (CE = 0.57), over *K* = 1 (CE = 0.51), based on their correspondence with PCA results, sampling distribution, and known geographic concentrations of northern bottlenose whales. Expected heterozygosity ranged from 0.170–0.177 and observed heterozygosity ranged from 0.172–0.184 (Table 1; Table S3), and no private alleles were present in any region. Estimates of differentiation (*F*_ST_) ranged from 0.002–0.020 (Table S4; Figure S6) and followed a strong pattern of IBD, with a significant correlation from the Mantel test *R* ^2^ of 0.86 (*p* = .032) (Figure 3a). Correlation of *F*_ST_ with latitude was weaker compared to distance, however it was still highly correlated with *R* ^2^ of 0.60 (*p* = .025) (Figure 3b). Given the influence of HWE filter methods on population structure (Pearman et al., 2022), we compared population structure results with and without the HWE filter and found minimal differences in our results, indicating the important SNPs defining population structure were retained.

### 3.4 Demographic history

**FIGURE 4:**
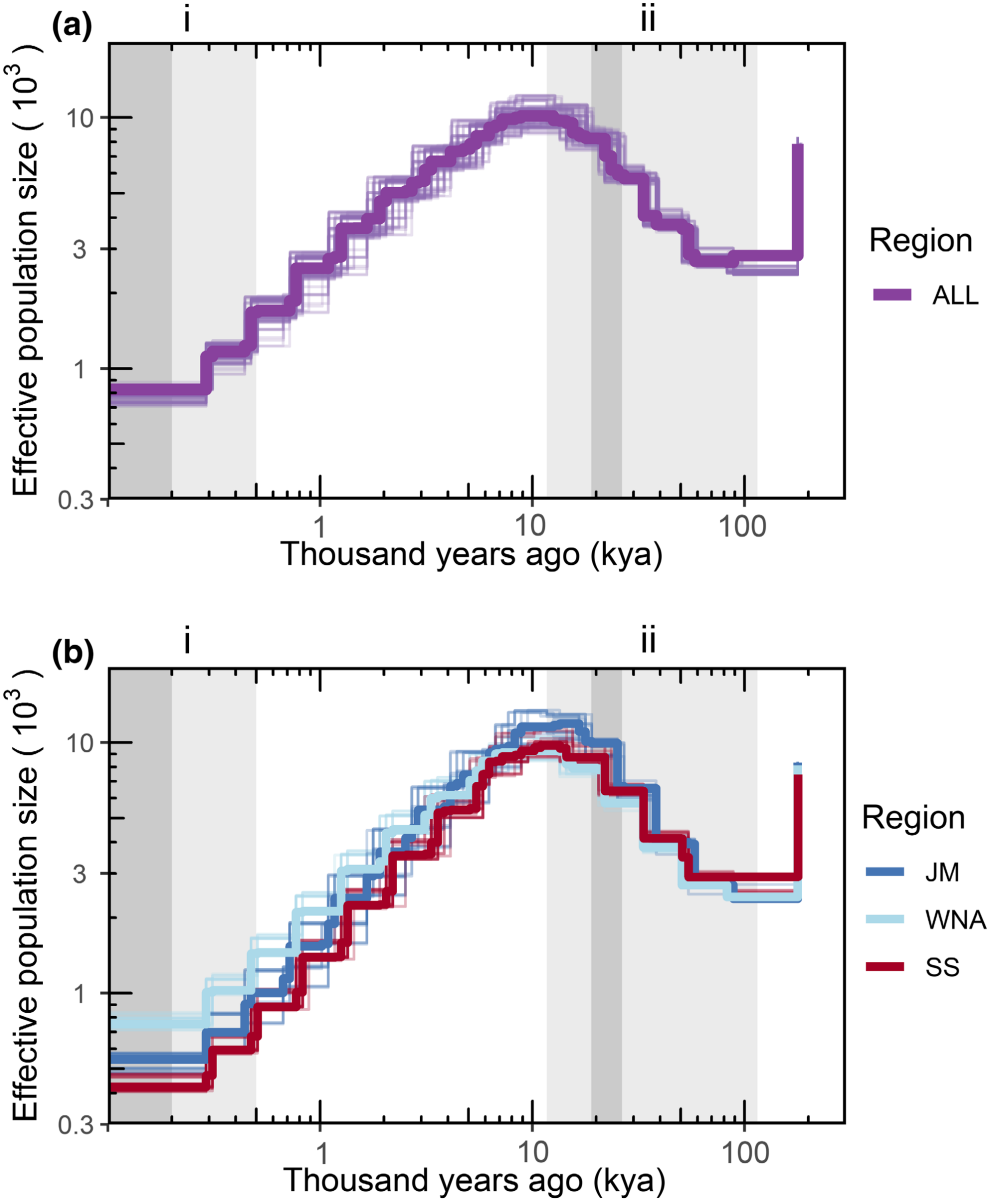
Demographic history of the northern bottlenose whale from 1,985,379 SNPs estimated as (a) one population and (b) three subgroups (JM, Jan Mayen; WNA, western North Atlantic; SS, Scotian shelf). Notable time periods are coloured in grey and marked by Roman numerals representing (i) the start of whaling in the North Atlantic, with dark grey bar representing large‐scale whaling in 19th–20th centuries, and (ii) last glacial period (11.7–115 kya) with dark grey bar representing last glacial maximum (19–26.5 kya). Plots were scaled with a mutation rate of 1.53 × 10^−8^ and generation time of 17.8 years.

The data set used in demographic history analyses was filtered down to 1,985,379 SNPs. We found that effective population size (*N*_e_) increased during the last glacial period (between 11.7–115 kya), including the last glacial maximum (between 19–26.5 kya), followed by a steady decline before commercial whaling began in the North Atlantic Ocean (Figure 4a). Models examining evolutionary history within subgroups presented high linkage across regions and suggests the declining trend in the Scotian Shelf population has exceeded the other regions within the last 500 years (Figure 4b).

### 3.5 Inbreeding

We used the same SNP set from population structure analyses, containing an LD‐pruned set of 995,508 SNPs. The number of ROHs and total length of ROHs were correlated (*r* = 1). Total ROH length was shorter in Jan Mayen (mean = 19.9 Mb; SD = 12.6 Mb; 95% CI: 9.4–30.4) compared to western North Atlantic (mean = 28.3 Mb; SD = 17.4 Mb; 95% CI: 20.2–36.4) and the Scotian Shelf (mean = 33.4 Mb; SD = 19.9 Mb; 95% CI: 18.1–48.7) (Table S5; Figure S7), however due to overlapping CIs, there was no significant difference between regions. The Scotian Shelf had the largest range in total ROH length (1.5–60.7 Mb), with some individuals containing long homozygous genotypes totalling up to 60 Mb, indicating individuals from this region had the highest metric of inbreeding in our study. These ROH estimates are limited to the degree of fragmentation in the reference assembly, and greater scaffold continuity could provide further details on ROH metrics.

### 3.6 Regions under selection

**FIGURE 5:**
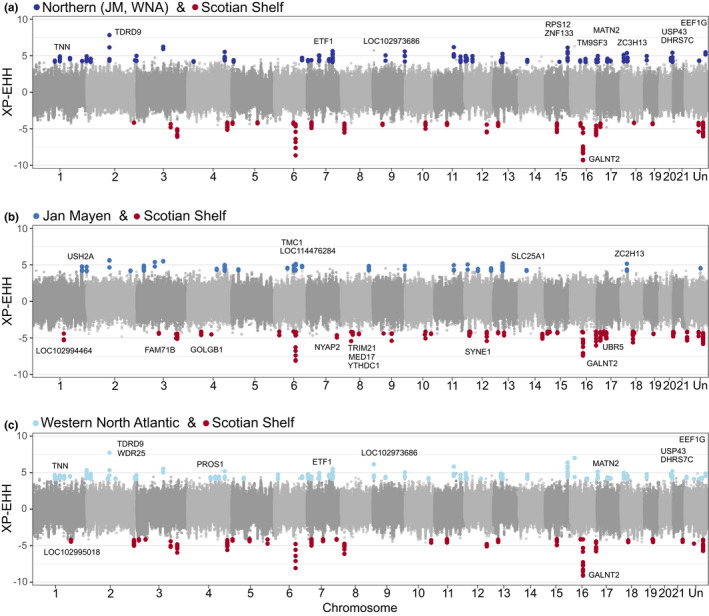
XP‐EHH in the northern bottlenose whale from 1,264,382 SNPs between (a) Scotian shelf and northern regions including Jan Mayen and western North Atlantic. The northern group was divided into subgroups for comparisons with Scotian shelf using (b) Jan Mayen only, and (c) western North Atlantic as one group. Candidate regions under selection are coloured blue in northern groups, and red in Scotian shelf, including genes within 20 kb windows of candidate regions.

The data set used in haplotype analyses was filtered down to 1,264,382 SNPs. XP‐EHH results revealed signatures of selection between the northern and Scotian Shelf populations (Figure 5a), indicating presence of multiple regions that may be associated with regional adaptations. Using these candidate regions, we located 12 genes within 20 kb of 206 significant SNPs in the northern populations, and eight genes within 20 kb of 111 significant SNPs in the Scotian Shelf population (Table S6). We excluded two genes (*PMM1* and *PSMD12*) that appeared in a high number of candidate regions that were over‐represented in the reference annotation, which may have resulted from annotating the genome without mRNA data, affecting the low confidence in locations of these two genes. GO analyses revealed weak enrichment in genes across the GO categories of biological processes, molecular function, and cellular components (Tables S7 and S8). By separating the northern region into Jan Mayen and western North Atlantic for comparisons with the Scotian Shelf (Figure 5b, c), we identified genomic regions that were only evident when comparing these groups separately, and genomic regions that were expressed in the Scotian Shelf in all three XP‐EHH analyses. Candidate regions expressed consistently in the Scotian Shelf were on chromosome 16 near the *GALNT2* gene, which may have a role in the biological processes of metabolism (Khetarpal et al., 2017) and insulin sensitivity (Marucci et al., 2013).

## 4 DISCUSSION

We examined population structure, demographic history, evidence of inbreeding, and signatures of selection in the northern bottlenose whale using whole‐genome resequencing data from five regions across the North Atlantic. Our results highlight genetic subdivision of the Scotian Shelf and Jan Mayen regions, and suggest subtle genetic variation within the western North Atlantic differentiating Canadian Arctic, Labrador, and Newfoundland regions. The observed long‐term decline in effective population size highlights the vulnerability of northern bottlenose whales to the negative effects of low diversity (Wade et al., 2012), and anthropogenic stressors, such as habitat degradation, behavioural impacts related to underwater noise (Wensveen et al., 2019), and climate change (Lambert et al., 2014; Record et al., 2019). The Scotian Shelf population, which is listed as Endangered under Canada's Species At Risk Act (COSEWIC, 2011), warrants priority for conservation action due to metrics indicative of inbreeding and evidence of local evolutionary adaptation.

Genomic analyses supported that the most prominent genetic subdivision is between the Scotian Shelf and northern regions, and revealed new evidence of genetic subdivision between Jan Mayen and western North Atlantic regions. This supports the identification of at least three northern bottlenose whale genetic subpopulations: the Scotian Shelf, western North Atlantic, and Jan Mayen, and corroborates previous population structure assessments using more limited microsatellite analyses (Einfeldt et al., 2022; Feyrer et al., 2019). The strong pattern of IBD highlights the importance of geographical distance in structuring genomic diversity in northern bottlenose whales and suggests that this species has limited connectivity between core habitats across its range. Our study captured additional signals of subtle structure within the western North Atlantic aligning with latitudinal clustering, consistent with IBD occurring within this broader habitat region (spanning 2375 km). Although the identification of subdivisions within the western North Atlantic was dominated by the Canadian Arctic (*n* = 11) and limited by small sample sizes from Labrador (*n* = 3) and Newfoundland (*n* = 6), the subtle variation found in these areas may be important given the species' low overall genetic diversity.

Declines in genetic diversity can impose risks to the persistence of wildlife populations, making species vulnerable to rapidly changing environments and pressures from human activity. Our results show that the northern bottlenose whale has experienced declines in effective population size since the last glacial period. While the subpopulations show similar trajectories, within the last 500 years, the lower effective population size in the Scotian Shelf population indicates a greater conservation concern. A steeper decline in the Scotian Shelf population was inferred from the analyses of mitogenomes in Feyrer et al. (2019). However, Feyrer et al. (2019) found a different pattern of expansion in the demographic history of the northern region. Differences between the results of Feyrer et al. (2019) results and ours may be due to different methods, genome coverage, sample sizes, sample locations, or inclusion of older whaled samples in Feyrer et al. (2019). However, given the maternal inheritance of mitogenomes, the findings of Feyrer et al. (2019) could also reflect the expansion of a northern matrilineal lineage after the last glacial period following changes in historically available habitat, ocean productivity, or connectivity between isolated populations. While we have a general understanding of northern bottlenose whale ecology and their preference for deep‐water prey species, further study is required to better understand the quantity and quality of habitat that would have been available during the last glacial period. Genomic patterns support the inference that the northern bottlenose whale expansion during the last glacial period was followed by a decline. In the deep‐diving Ramari's beaked whale, the historical effective population size also expanded during the last glacial period (Carroll et al., 2021). While some marine mammal species exhibit the opposite trend compared to the northern bottlenose whale, with an increasing effective population size after the last glacial period (Cabrera et al., 2022), variation among trajectories in species inhabiting the North Atlantic Ocean include other species that also present a declining trend after the last glacial period (Cabrera et al., 2022). Other signals of regional differences in the northern bottlenose whale population history were not detected, but may have been masked by historical gene flow. The long‐term decline in effective population size probably made the species more vulnerable to the impacts of commercial whaling in the 19th–20th century, and exacerbated risks associated with the loss of genetic diversity.

In the Scotian Shelf population, the longer values of ROH (i.e., when compared with Jan Mayen), combined with a possible greater demographic decline, indicate a higher risk of negative genetic effects, such as inbreeding depression. In Jan Mayen, lower mean values of ROH, could reflect a larger population size relative to other parts of the species' range. ROH values indicate the highest degree of inbreeding comes from individuals in the Scotian Shelf, although the range of values overlaps those found across the western North Atlantic. While an assembly with higher continuity could improve ROH estimates, these results are consistent with predictions in smaller populations. For example, killer whale populations that have gone through population bottlenecks exhibit higher ROH estimates (Foote et al., 2021). Observed heterozygosity metrics are similar across populations; however, the initial effect of inbreeding may be seen in the reduced fitness due to homozygosity (Barrett & Charlesworth, 1991), preceding changes in levels of heterozygosity. ROH provides additional information about how the diversity that is present in populations is distributed at the individual level. Higher ROH within some individuals in the Scotian Shelf suggest a higher degree of inbreeding in this region. As inbreeding can impose health risks to populations, these signals of inbreeding emphasize the need to prioritize conservation of core areas and habitat connectivity between and within regions, since the loss of genetic diversity in an inbred group may limit opportunities for adaptation (Neaves et al., 2015).

Unique conditions in different geographic regions, particularly latitudinal variation in temperature and other climatic factors, can lead to different selective pressures. Signatures of recent positive selection in a cross‐population analysis between the Scotian Shelf and northern regions suggest evolutionary processes are locally structured between these groups. Although we did not find significant genes sharing functional characteristics, there were strong genomic regions of selection consistently represented in the Scotian Shelf. This occurred both when compared to the northern region as a whole, and when compared to Jan Mayen and the western North Atlantic separately. One of these regions was near *GALNT2*, a gene with roles in metabolism, lipid regulation (Khetarpal et al., 2017), and insulin sensitivity (Marucci et al., 2013), suggesting potentially different selective pressure(s) on diet and metabolism, such as warmer climates in southern regions. Miller et al. (2016) found consistent and substantial differences in estimated quantities of lipid stores between whales tagged near Jan Mayen and individuals from the Scotian Shelf (Miller et al., 2016). However, inferences about the functionality of northern bottlenose whale adaptations should be considered putative and interpreted with caution until tested more explicitly.

## 5 CONCLUSION

This study provides a foundation for northern bottlenose whale genomic research and expands our understanding of beaked whale evolution across the North Atlantic. Our results indicate subgroups contain distinct genomic attributes, defining three prominent demographic units for northern bottlenose whales: the Scotian Shelf, western North Atlantic, and Jan Mayen. The pattern of isolation‐by‐distance suggests dispersal among regions across their range is limited, highlighting the need to conserve connectivity these populations have experienced in their evolutionary past. Given that adaptation occurs even when gene flow exists, it is important to maintain existing levels of genetic diversity available for this species to respond to external threats. At the southern edge of their range, isolated from other regions, with a smaller census and effective population size, and exhibiting elevated signals of inbreeding, the Scotian Shelf population is likely to be at greater risk from contemporary threats than northern bottlenose whales in other regions. Reducing threats to individual survival and reproduction is necessary to rebuild small populations. However, preserving unique adaptive diversity also requires protecting habitat connectivity throughout the species' range to help mitigate the negative impacts of long‐term population declines and isolation.

## AUTHOR CONTRIBUTIONS

Laura J. Feyrer, Anthony L. Einfeldt, and Paul Bentzen designed and supervised this study; Evelien de Greef and Anthony L. Einfeldt conducted formal analyses; Laura J. Feyrer, Anthony L. Einfeldt, Paul Bentzen, Steven H. Ferguson, Patrick J. O. Miller, and Evelien de Greef acquired funding; Laura J. Feyrer, Patrick J. O. Miller, and Kyle Lefort collected tissue samples; Ian G. Paterson conducted laboratory protocols; Colin J. Garroway provided conceptual input and analytical tools; Evelien de Greef, Laura J. Feyrer, and Anthony L. Einfeldt wrote the manuscript with input from all authors.

## CONFLICT OF INTEREST

The authors declare no conflicting interests.

### OPEN RESEARCH BADGES

This article has earned an Open Data badge for making publicly available the digitally‐shareable data necessary to reproduce the reported results. The data is available on Zenodo (10.5281/zenodo.6640042) and in BioProject PRJNA809035.

## Supporting information


Appendix S1

## Data Availability

Genotype data have been made available on Zenodo (10.5281/zenodo.6640042; de Greef et al., 2022) and scripts are available on github (github.com/edegreef/NBW‐resequencing). Raw sequence data and reference genome are deposited to NCBI database (BioProject PRJNA809035).
