# Supplementary material for: Genomics reveal population structure, evolutionary history, and signatures of selection in the northern bottlenose whale, Hyperoodon ampullatus
Source: Mol Ecol. 2022 Aug 23;31(19):4919–31. doi: 10.1111/mec.16643 (PMC9804413; doi:10.1111/mec.16643)
Supplement: Supplementary file 1 — Appendix S1 [file MEC-31-4919-s001.pdf]

MOLECULAR ECOLOGY

Supplemental Information for:

Genomics reveal population structure, evolutionary history, and signatures of selection in the northern bottlenose whale, *Hyperoodon ampullatus*

Evelien de Greef, Anthony L. Einfeldt, Patrick J. O. Miller, Steven H. Ferguson,

Colin J. Garroway, Kyle J. Lefort, Ian G. Paterson, Paul Bentzen, and Laura J. Feyrer

Table of Contents:

**Methods**	Page 2
**Reference genome**	Page 2
**Resequencing data**	Page 3
**References**	Page 5
**Tables and Figures**	Page 7
**Table S1 – reference genome statistics**	Page 7
**Table S2 – resequencing data statistics**	Page 8
**Table S3 – diversity indices summary**	Page 10
**Table S4 – pairwise estimates of F_{ST} summary**	Page 11
**Table S5 – runs of homozygosity summary**	Page 12
**Table S6 – genes within 20 kb of regions under selection**	Page 13
**Table S7 – grouped gene ontology enrichment results**	Page 14
**Table S8 – separated gene ontology enrichment results**	Page 15
**Figure S1 – proportion of chromosome lengths with blue whale**	Page 17
**Figure S2 – SNP filtering step**	Page 18
**Figure S3 – SNP metrics**	Page 19
**Figure S4 – coverage of sex-linked scaffolds**	Page 20
**Figure S5 – PCAs with sex-linked SNPs**	Page 21
**Figure S6 – heatmap of pairwise estimates of F_{ST}**	Page 22
**Figure S7 – runs of homozygosity**	Page 23

MOLECULAR ECOLOGY

Reference Genome

Synteny

We mapped the northern bottlenose whale (*Hyperoodon ampullatus*) genome to the blue whale (*Balaenoptera musculus*) to determine chromosomal positions with *SatsumaSynteny* v2 (Grabherr et al., 2010). Due to the large genome size (>1.5 Gb), we ran *SatsumaSynteny* for each blue whale chromosome separately. We then filtered out scaffold alignments with an identity value below 0.6 and scaffolds with less than 50% of their length mapped to a chromosome.

Annotation

Genome annotation through *MAKER* v2.31.10 (Holt & Yandell, 2011) required multiple iterations to train *SNAP* (computational gene prediction) and create hidden Markov models (HMM) to improve annotations. In the first round, we generated gene models using protein data obtained from *Ensembl* database (Cunningham et al., 2019) for three model species: blue whale, sperm whale (*Physeter macrocephalus*), and cow (*Bos taurus*). We adjusted parameters for masking repeats with RepeatMasker (model_org=all) and aligning splice sites for protein sequences with Exonerate (protein2genome=1). In the second round, we used the generated HMM results from the first round as an input parameter for *SNAP* to update the annotation. We repeated this process in the third round, including the parameter for additional gene prediction with *Augustus* (augustus_species=human). In the fourth round, we updated the annotation with the previous round's HMM results for a final time, then filtered out annotations with an annotation edit distance (AED) greater than 0.5. We then aligned the northern bottlenose whale annotation with protein data with the same three model organisms (blue whale, sperm whale, cow) from uniprot database (The UniProt Consortium, 2019) using *BLAST+* v2.11.0 (Camacho et al., 2009) and protein functions using *InterProScan* v5.23-62.0 (Jones et al., 2014), providing gene identifications and gene ontology (GO) information.

MOLECULAR ECOLOGY

Resequencing data

Merging sequence data for two individuals

We had duplicate sequencing reads for some of our individuals (due to a laboratory error, 24 samples were re-sequenced). To recover some individuals that were removed from the SNP dataset due to high missingness, we identified sample matches and merged the sequencing data to increase sample completeness. In this process, SNPs were called with *Platypus* v0.8.1 (Rimmer et al., 2014), using a total of 73 bam files (the original 49 and re-sequenced 24 samples). Then we ran a kinship analysis with *PLINK* v1.9 (Purcell et al., 2007) to determine duplicates. Through this method, we were able to confidently identify which sequencing reads to merge for two individuals, allowing us to retain these two samples that would have been filtered out from our main dataset otherwise. We merged aligned bam files with *SAMtools* v1.9 (Li et al., 2009) and redid deduplication and read group addition for these two samples with *Picard* v2.20.6 (Broad Institute, 2019). These updated bam files were included in SNP calling for downstream analyses.

Sex determination

Sex chromosomes in mammals have large areas that are non-recombining, and differences in sex chromosomes between males (XY) and females (XX) may drive a proportion of genetic variation among individuals and bias inferences of evolutionary processes that assume recombination occurs. To isolate autosomes from sex chromosomes for downstream analyses, we identified genomic regions with scaffolds exhibiting differences in coverage consistent with being on the X and Y chromosomes. We compared sequencing depth in windows across the genome between male and female samples with *DifCover* (Smith et al., 2018), using bam files aligned to the reference genome. We used two samples from each sex to run four experimental comparisons with different-sex samples and two control comparisons with same-sex samples (Grayson et al., 2022). *BEDTools* v2.25.0 (Quinlan & Hall, 2010) was used to compare results from each run and list genomic regions that were consistently different in coverage between males and females and consistently similar in coverage in same-sex comparisons, identifying scaffolds located on the X and Y chromosomes.

Structural variants

Through the program *BreakDancer* v1.3.6 (Fan et al. 2014), we used 10 samples (bam files aligned to the reference genome) with 8-10x coverage to identify structural variants, containing inter-chromosomal translocations, intra-chromosomal translocations, and inversions. We selected structural variants with confidence scores above 50, then created a list of all SNPs that fell in these regions to use in filtering the SNP dataset.

Linkage disequilibrium decay

Alleles found together at a greater frequency compared to random assortment are described to be in linkage disequilibrium (LD), where greater r^2 indicates higher linkage

disequilibrium (Slatkin, 2009). Generally, LD decay exhibits a decrease in r^2 with increasing inter-SNP distance. We used *PopLDdecay* to complete a LD decay analysis directly on the SNP vcf file (Zhang et al., 2019). After observing a bump in the LD decay plot that did not align with expected LD, we predicted that small scaffolds may be influencing results. Accordingly, we removed SNPs on scaffolds shorter than 50 kb, which resulted in more accurate LD decay estimates. Given impact of these short scaffolds, we excluded them from downstream analyses.

References

Broad Institute. 2019 Picard Toolkit. GitHub Repository. http://broadinstitute.github.io/picard/

Camacho, C., Coulouris, G., Avagyan, V., Ma, N., Papadopoulos, J., Bealer, K., & Madden, T. L. (2009). BLAST+: architecture and applications. *BMC Bioinformatics, 10*, 421. https://doi.org/10.1186/1471-2105-10-421

Cunningham, F., Achuthan, P., Akanni, W., Allen, J., Amose, M. R., Armean, I. M., Bennett, R., Bhai, J., Billis, K., Boddu, S., Cummins, C., Davidson, C., Dodiya, K. J., Gall, A., Girón, C. G., Gil, L., Grego, T., Haggerty, L., Haskell, E., … Flicek, P. (2019). Ensembl 2019. *Nucleic Acids Research*, *47*, D745–D751. https://doi.org/10.1093/nar/gky1113

Fan, X., Abbot, T. E., Larson, D., & Chen, K. (2014). BreakDancer: identification of genomic structural variation from paired-end read mapping. *Current Protocols in Bioinformatics, 45*, 15.6.1–15.6.11. https://doi.org/10.1002/0471250953.bi1506s45

Grabherr, M. G., Russell, P., Meyer, M., Mauceli, E., Alföldi, J., Di Palma, F., & Lindblad-Toh, K. (2010). Genome-wide synteny through highly sensitive sequence alignment: *Satsuma*. *Bioinformatics, 26*(9), 1145–1151. https://doi.org/10.1093/bioinformatics/btq102

Grayson, P., Wright, A., Garroway, C. J., & Docker, M. F. (2022). SexFindR: A computational workflow to identify young and old sex chromosomes. *BioRxiv.* https://doi.org/10.1101/2022.02.21.481346

Holt, C., & Yandell, M. (2011). MAKER2: an annotation pipeline and genome-database management tool for second-generation genome projects. *BMC Bioinformatics, 12,* 491. https://doi.org/10.1186/1471-2105-12-491

Jones, P., Binns, D., Chang, H. Y., Fraser, M., Li, W., McAnulla, C., McWilliam, H., Maslen, J., Mitchell, A., Nuka, G., Pesseat, S., Quinn, A. F., Sangrador-Vegas, A., Scheremetjew, M., Yong, S. Y., Lopez, R., & Hunter, S. (2014). InterProScan 5: genome-scale protein function classification. *Bioinformatics, 30*(9), 1236–1240. https://doi.org/10.1093/bioinformatics/btu031

Li, H., Handsaker, B., Wysoker, A., Fennell, T., Ruan, J., Homer, N., Gabor, M., Abecasis, G., Durbin, R., & 1000 Genome Project Data Processing Subgroup. (2009). The sequence alignment/map format and SAMtools. *Bioinformatics, 25*(16), 2078–2079. https://doi.org/10.1093/bioinformatics/btp352

MOLECULAR ECOLOGY

Purcell, S., Neale, B., Todd-Brown, K., Thomas, L., Ferreira, M. A. R., Maller, B. D., Sklar, P., de Bakker, P. I. W., Daly, M. J., & Sham, P. C. (2007). PLINK: a toolset for whole-genome association and population-based linkage analysis. *American Journal of Human Genetics, 81*(3), 559–575. https://doi.org/10.1086/519795

Quinlan, A. R., & Hall, I. M. (2010). BEDTools: a flexible suite of utilities for comparing genomic features. *Bioinformatics, 26*(6), 841–842. https://doi.org/10.1093/bioinformatics/btq033

Rimmer, A., Phan, H., Mathieson, I., Iqbal, Z., Twigg, S. R. F., WGS500 Consortium, Wilkie, A. O. M., McVean, G., & Lunter, G. (2014). Integrating mapping-, assembly- and haplotype-based approaches for calling variants in clinical sequencing applications. *Nature Genetics, 46*, 912–918. https://doi.org/10.1038/ng.3036

Slatkin, M. (2009). Linkage disequilibrium —understanding the evolutionary past and mapping the medical future. *Nature Reviews Genetics, 9*, 477– 485. https://doi.org/10.1038/nrg2361

Smith, J.J., Timoshevskaya, N., Ye, C., Holt, C., Keinath, M. C., Parker, H. J., Cook, M. E., Hess, J. E., Narum, S. R., Lamanna, F., Kaessmann, H., Timoshevskiy, V. A., Waterbury, C. K. M., Saraceno, C., Wiedemann, L. M., Robb, S. M. C., Baker, C., Eichler, E. E., Hockman, D., Sauka-Spengler, T., Yandell, M., Krumlauf, R., Elgar, G., & Amemiya, C. T. (2018). The sea lamprey germline genome provides insights into programmed genome rearrangement and vertebrate evolution. *Nature Genetics, 50*, 270–277. https://doi.org/10.1038/s41588-017-0036-1

The UniProt Consortium. (2019). UniProt: a worldwide hub of protein knowledge. *Nucleic Acids Research, 47*(D1)*,* D506–D515. https://doi.org/10.1093/nar/gky1049

Zhang, C., Dong, S. S., Xu, J. Y., He, W. M., & Yang, T. L. (2019). PopLDdecay: a fast and effective tool for linkage disequilibrium decay analysis based on variant call format files. *Bioinformatics, 35*(10), 1786–1788. https://doi.org/10.1093/bioinformatics/bty875

MOLECULAR ECOLOGY

Tables & Figures

Table S1. Summary statistics of northern bottlenose whale reference genome assembly.

**Reference genome metrics**	
Genome size (bp)	2,353,367,117
Number of scaffolds	67,191
Number of scaffolds >50kb (% genome)	13,062 (80%)
Number of scaffolds >100kb (% genome)	5,983 (59%)
Number of scaffolds >1Mb (% genome)	39 (2%)
Longest scaffold (bp)	2,341,931
Shortest scaffold (bp)	1,000
Mean scaffold length (bp)	35,025
N50 scaffold length (bp)	132,959
Number of contigs	109,366
N50 contig length (bp)	60,024
GC content (%)	41.16
N's per 100 kb	900
Complete BUSCOs	7,004 (75.9%)
Complete single-copy BUSCOs	6,902 (74.8%)
Complete duplicated BUSCOs	102 (1.1%)
Fragmented BUSCOs	743 (8.1%)
Missing BUSCOs	1,479 (16.0%)

MOLECULAR ECOLOGY

Table S2. Summary statistics of northern bottlenose whale resequencing data, including number of reads, mean and modal coverage, coverage of down-sampled individuals, frequency of SNP missingness per individual, and noting which samples were removed from analyses due to high missingness (MISS), duplicate (DUP), or kin pair (KIN), with labels for each DUP and KIN pair and which individual from each pair was retained ("kept") or removed ("rem.").

Sample ID	Region	Raw reads	Mapped reads	Mean coverage	Modal coverage	Down-sampled modal coverage	SNP freq. miss.	Removed (MISS, DUP, KIN)
BKW1328	Jan Mayen	134,920,022	133,913,350	8.3	4		0.132	DUP1 – kept
BKW1330	Jan Mayen	150,082,702	148,939,363	9.3	4		0.172	
BKW1331	Jan Mayen	84,647,705	84,064,269	5.8	2		0.302	
BKW1332	Jan Mayen	59,857,411	59,394,699	4.6	1		0.431	MISS – rem.
BKW1334	Jan Mayen	197,630,078	196,347,787	12.4	7	4	0.149	DUP1 – rem.
BKW1335	Jan Mayen	172,712,556	171,629,754	10.8	5		0.115	
BKW1340	Jan Mayen	314,933,068	313,224,629	19.1	10	4	0.125	
BKW1343	Jan Mayen	44,737,608	44,446,574	3.9	1		0.532	MISS – rem.
BKW1345	Jan Mayen	319,462,318	316,949,285	10.2	11	4	0.198	
BKW1333	Jan Mayen	181,626,721	180,436,880	10.2	6		0.169	
BKW1337	Jan Mayen	97,652,242	97,138,170	6.2	2		0.231	
BKW1017	Canadian Arctic	122,592,829	121,517,927	7.4	5		0.143	
BKW1018	Canadian Arctic	200,302,990	199,092,838	11.6	8	5	0.103	
BKW1019	Canadian Arctic	187,676,475	186,726,297	10.9	8	4	0.124	
BKW1020	Canadian Arctic	194,379,733	192,921,195	11.4	8	5	0.102	
BKW1312	Canadian Arctic	180,168,062	178,775,934	11.3	5		0.108	DUP2 – kept
BKW1313	Canadian Arctic	278,886,765	276,665,426	16.6	10	4	0.164	DUP3 – kept
BKW1314	Canadian Arctic	130,704,906	129,853,815	8.1	4		0.142	DUP2 – rem.
BKW1315	Canadian Arctic	272,984,426	271,225,752	16.2	10	4	0.136	
BKW1316	Canadian Arctic	2991,22,793	297,316,465	17.6	11	4	0.146	
BKW1317	Canadian Arctic	62,929,318	62,387,652	4.5	1		0.333	
BKW1318	Canadian Arctic	133,273,318	132,062,778	8.3	3		0.196	
BKW1319	Canadian Arctic	181,660,019	180,416,300	11.2	6	5	0.126	
BKW1305	Canadian Arctic	202,198,682	200,603,994	12.9	1		0.133	DUP3 – rem.
BKW0360	Labrador	245,493,065	243,625,863	14.2	9	5	0.128	
BKW0359	Labrador	260,825,528	258,366,901	14.9	10	5	0.083	
BKW0358	Labrador	249,233,669	246,652,708	14.6	11	4	0.095	
Hyam-2016-06	Newfoundland	169,842,711	168,656,097	10.2	7	5	0.122	DUP4 – rem.
BKW0492	Newfoundland	151,615,349	148,711,007	9.1	6	5	0.089	
BKW0484	Newfoundland	44,840,452	44,531,211	3.4	1		0.500	MISS – rem.
BKW0489	Newfoundland	216,262,100	214,191,575	12.9	9	5	0.136	DUP4 - kept
BKW0454	Newfoundland	56,821,731	56,220,438	3.9	2		0.310	
BKW0456	Newfoundland	215704935	213,960,372	12.5	9	4	0.090	
BKW0451	Newfoundland	24,543,025	24,148,358	2.3	1		0.619	MISS – rem.
BKW0452	Newfoundland	122,847,289	121,673,304	7.4	5		0.098	
BKW0453	Newfoundland	87,852,763	87,078,560	5.5	3		0.219	
BKW0480	Scotian Shelf	229,103,623	226,874,856	13.7	10	5	0.093	
BKW0475	Scotian Shelf	113,054,911	112,161,973	6.9	4		0.118	KIN1 – rem.

BKW0466	Scotian Shelf	164,754,988	163,175,740	9.9	7	5	0.138	
BKW0458	Scotian Shelf	25,195,037	24,962,236	2.5	1		0.654	MISS – rem.
BKW0477	Scotian Shelf	144,433,434	143,324,589	8.8	5		0.123	
BKW0474	Scotian Shelf	134,177,460	132,921,377	8.1	6	5	0.096	KIN1 – kept
BKW0461	Scotian Shelf	26,495,737	26,107,343	2.4	1		0.591	MISS – rem.
BKW0955	Scotian Shelf	71,059,132	70,646,024	4.7	2		0.312	
BKW0956	Scotian Shelf	57,9937	408,921	1.5	1		0.996	MISS – rem.
BKW0958	Scotian Shelf	173,877,629	172,388,296	10.7	6	5	0.164	
BKW0960	Scotian Shelf	147,454,822	146,417,233	9.1	4		0.170	
BKW0957	Scotian Shelf	236,043,322	234,419,044	14.5	9	4	0.191	
BKW0962	Scotian Shelf	172,316,862	171,018,186	10.5	5		0.149	

MOLECULAR ECOLOGY

Table S3. Summary results of diversity indices in the northern bottlenose whale for expected heterozygosity (H_E) and observed heterozygosity (H_O)

Region	H_E mean	H_E lower 95 CI	H_E upper 95 CI	H_O mean	H_O lower 95 CI	H_O upper 95 CI
Jan Mayen	0.176	0.175	0.176	0.179	0.178	0.179
Canadian Arctic	0.176	0.175	0.176	0.177	0.177	0.178
Labrador	0.170	0.170	0.171	0.172	0.172	0.173
Newfoundland	0.177	0.177	0.177	0.184	0.183	0.184
Scotian Shelf	0.174	0.174	0.174	0.178	0.178	0.179

MOLECULAR ECOLOGY

Table S4. Summary of pairwise estimates of differentiation in the northern bottlenose whale estimated through Reich's F_{ST}. Pair abbreviations are: JM=Jan Mayen, CA=Canadian Arctic, LB=Labrador, NF=Newfoundland, LB=Labrador.

Pair	F_{ST}	Lower 95 CI	Upper 95 CI	P-value
CA-JM	0.00829	0.00795	0.00864	less than 2e-16
CA-LB	0.00311	0.00273	0.00349	less than 2e-16
CA-NF	0.00337	0.00303	0.00372	less than 2e-16
CA-SS	0.01270	0.01237	0.01302	less than 2e-16
JM-LB	0.01384	0.01343	0.01425	less than 2e-16
JM-NF	0.01288	0.01251	0.01325	less than 2e-16
JM-SS	0.02043	0.02008	0.02078	less than 2e-16
LB-NF	0.00159	0.00119	0.00199	5.77E-15
LB-SS	0.01427	0.01389	0.01466	less than 2e-16
NF-SS	0.01218	0.01183	0.01259	less than 2e-16

MOLECULAR ECOLOGY

Table S5. Summary results of runs of homozygosity (ROH) analyses across three northern bottlenose whale groups: Jan Mayen, Western North Atlantic, and Scotian Shelf.

	Jan Mayen	Western North Atlantic	Scotian Shelf
**whale samples (*n*)**	8	20	9
**Total ROH mean (Mb)**	19.86	28.31	33.40
**Total ROH lower 95 CI (Mb)**	9.36	29.20	18.13
**Total ROH upper 95 CI (Mb)**	30.36	36.43	48.67
**Total ROH SD (Mb)**	12.56	17.34	19.86
**Number of ROH > 100 kb**	800	2984	1565
**Number of ROH > 250 kb**	16	114	75
**Number of ROH > 500 kb**	0	1	2
**Number of ROH > 1 Mb**	0	0	0

MOLECULAR ECOLOGY

Table S6. List of genes within 20 kb of regions under selection identified through XP-EHH analyses in the northern bottlenose whale.

Chr	Gene	Description
Northern (JM, WNA)		
1	TNN	Tenascin-N isoform X2
2	TDRD9	ATP-dependent RNA helicase TDRD9
7	ETF1	Eukaryotic peptide chain release factor subunit 1
9	LOC102973686	Dipeptidase
15	RPS12	40S ribosomal protein S12
15	ZNF133	Zinc finger protein 133 isoform X1
16	TM9SF3	Transmembrane 9 superfamily member
17	MATN2	Matrilin-2 isoform X6 (Physeter macrocephalus
18	ZC3H13	Zinc finger CCCH domain-containing protein 13 isoform X1
20	USP43	Ubiquitin carboxyl-terminal hydrolase 43 isoform X1
20	DHRS7C	Dehydrogenase/reductase SDR family member 7C isoform X1
Un	EEF1G	Elongation factor 1-gamma
Jan Mayen		
1	USH2A	Usherin
6	TMC1	Transmembrane channel-like protein
6	LOC114487284	Nuclear pore-associated protein 1-like
14	SLC25A1	Tricarboxylate transport protein
18	ZC2H13	Zinc finger CCCH domain-containing protein 13 isoform X1
Western North Atlantic		
1	TNN	Tenascin-N isoform X2
2	TDRD9	ATP-dependent RNA helicase TDRD9
2	WDR25	WD repeat-containing protein 25
4	PROS1	Vitamin K-dependent protein S
7	ETF1	Eukaryotic peptide chain release factor subunit 1
9	LOC102973686	Dipeptidase
17	MATN2	Matrilin-2 isoform X6
20	USP43	Ubiquitin carboxyl-terminal hydrolase 43 isoform X1
20	DHRS7C	Dehydrogenase/reductase SDR family member 7C isoform X1
Un	EEF1G	Elongation factor 1-gamma
Scotian Shelf		
1	LOC102995018	Protein transport protein sec16
1	LOC102994464	Myomegalin
3	FAM71B	Protein FAM71B
4	GOLGB1	Golgin subfamily B member 1 isoform X5
7	NYAP2	Neuronal tyrosine-phosphorylated phosphoinositide-3-kinase adapter 2
7	NA	60S ribosomal protein L17
8	TRIM21	E3 ubiquitin-protein ligase TRIM21
8	MED17	Mediator of RNA polymerase II transcription subunit 17
8	YTHDC1	YTH domain-containing protein 1 isoform X2
8	NA	Gag_p30 domain-containing protein
12	SYNE1	Nesprin-1 isoform X2
16	GALNT2	Polypeptide N-acetylgalactosaminyltransferase
17	UBR5	E3 ubiquitin-protein ligase UBR5 isoform X5

MOLECULAR ECOLOGY

Table S7. Gene ontology enrichment results (adjusted p-value < 0.05) in candidate regions identified through XP-EHH analyses in the northern bottlenose whale comparing northern and Scotian shelf population haplotypes.

Region	Description	GO ID	Adjusted p-value	Combined score	Genes
Biological Process					
Northern	negative regulation of osteoblast proliferation	GO:0033689	0.049	1497	TNN
Northern	osteoblast development	GO:0002076	0.049	1251	TNN
Northern	male meiosis I	GO:0007141	0.049	932	TDRD9
Northern	cellular protein complex disassembly	GO:0043624	0.049	602	ETF1
Northern	translation	GO:0006412	0.049	73	EEF1G, ETF1
Northern	regulation of protein complex disassembly	GO:0043244	0.049	551	ETF1
Northern	regulation of osteoblast proliferation	GO:0033688	0.049	507	TNN
Northern	regulation of transposition	GO:0010528	0.049	469	TDRD9
Northern	piRNA metabolic process	GO:0034587	0.049	436	TDRD9
Northern	negative regulation of transposition	GO:0010529	0.049	407	TDRD9
Northern	regulation of neuron migration	GO:2001222	0.049	407	TNN
Northern	meiosis I	GO:0007127	0.050	358	TDRD9
Scotian Shelf	macromolecule biosynthetic process	GO:0009059	0.012	3253	GALNT2
Scotian Shelf	protein O-linked glycosylation via threonine	GO:0018243	0.012	2877	GALNT2
Scotian Shelf	protein O-linked glycosylation via serine	GO:0018242	0.012	2718	GALNT2
Scotian Shelf	oligosaccharide biosynthetic process	GO:0009312	0.012	1656	GALNT2
Scotian Shelf	carbohydrate biosynthetic process	GO:0016051	0.012	1489	GALNT2
Scotian Shelf	oligosaccharide metabolic process	GO:0009311	0.01	750	GALNT2
Scotian Shelf	O-glycan processing	GO:0016266	0.015	722	GALNT2
Scotian Shelf	peptidyl-threonine modification	GO:0018210	0.018	4889	GALNT2
Scotian Shelf	protein O-linked glycosylation	GO:0006493	0.021	355	GALNT2
Scotian Shelf	protein glycosylation	GO:0006486	0.021	304	GALNT2
Scotian Shelf	peptidyl-serine modification	GO:0018209	0.025	216	GALNT2
Molecular Function					
Northern	translation factor activity, RNA binding	GO:0008135	0.018	395	EEF1G, ETF1
Scotian Shelf	polypeptide N-acetylgalactosaminyltransferase activity	GO:0004653	0.010	2877	GALNT2
Scotian Shelf	manganese ion binding	GO:0030145	0.010	1541	GALNT2
Scotian Shelf	acetylgalactosaminyltransferase activity	GO:0008376	0.010	1048	GALNT2
Cellular Component					
Northern	germ plasm	GO:0060293	0.031	663	TDRD9
Northern	P granule	GO:0043186	0.031	507	TDRD9
Scotian Shelf	Golgi stack	GO:0005795	0.015	2024	GALNT2
Scotian Shelf	integral component of Golgi membrane	GO:0030173	0.015	1310	GALNT2

MOLECULAR ECOLOGY

Table S8. Gene ontology enrichment results (adjusted p-value < 0.05) in candidate regions identified through XP-EHH analyses in the northern bottlenose whale comparing Jan Mayen (JM) and Scotian Shelf (SS) population haplotypes, and western North Atlantic (WNA) and Scotian Shelf populations.

Region	Description	GO ID	Adjusted p-value	Combined score	Genes
Biological Process					
JM	inner ear receptor cell differentiation	GO:0060113	0.020	6500	USH2A
JM	hair cell differentiation	GO:0035315	0.020	3327	USH2A
JM	acyl-CoA biosynthetic process	GO:0071616	0.020	2174	SLC25A1
JM	fatty-acyl-CoA metabolic process	GO:0035337	0.020	1485	SLC25A1
JM	fatty acid derivative biosynthetic process	GO:1901570	0.020	1111	SLC25A1
JM	fatty-acyl-CoA biosynthetic process	GO:0046949	0.021	843	SLC25A1
JM	gluconeogenesis	GO:0006094	0.023	572	SLC25A1
JM	hexose biosynthetic process	GO:0019319	0.023	524	SLC25A1
JM	sensory perception	GO:0007600	0.024	405	USH2A
JM	glucose metabolic process	GO:0006006	0.024	328	SLC25A1
JM	epidermal cell differentiation	GO:0009913	0.024	309	USH2A
JM	sensory perception of mechanical stimulus	GO:0050954	0.024	255	USH2A
JM	sensory perception of sound	GO:0007605	0.024	243	USH2A
JM	sensory perception of light stimulus	GO:0050953	0.024	243	USH2A
JM	mitochondrial transport	GO:0006839	0.036	126	SLC25A1
JM	neuron differentiation	GO:0030182	0.036	121	USH2A
JM	negative regulation of cell differentiation	GO:0045596	0.037	110	USH2A
WNA	peptide metabolic process	GO:0006518	0.045	284	EEF1G, PROS1
WNA	negative regulation of osteoblast proliferation	GO:0033689	0.045	1852	TNN
WNA	osteoblast development	GO:0002076	0.045	1549	TNN
WNA	male meiosis I	GO:0007141	0.045	1156	TDRD9
WNA	peptidyl-glutamic acid carboxylation	GO:0017187	0.045	1021	PROS1
WNA	protein carboxylation	GO:0018214	0.045	1021	PROS1
WNA	Translation	GO:0006412	0.045	95	EEF1G, ETF1
WNA	cellular protein complex disassembly	GO:0043624	0.045	748	ETF1
WNA	regulation of protein complex disassembly	GO:0043244	0.045	685	ETF1
WNA	regulation of osteoblast proliferation	GO:0033688	0.045	631	TNN
WNA	regulation of transposition	GO:0010528	0.045	584	TDRD9
WNA	piRNA metabolic process	GO:0034587	0.045	543	TDRD9
WNA	negative regulation of transposition	GO:0010529	0.045	507	TDRD9
WNA	regulation of neuron migration	GO:2001222	0.045	507	TNN
WNA	meiosis I	GO:0007127	0.046	446	TDRD9

MOLECULAR ECOLOGY

WNA	regulation of release of sequestered calcium ion into cytosol by sarcoplasmic reticulum	GO:0010880	0.047	397	DHRS7C
WNA	signal peptide processing	GO:0006465	0.047	357	PROS1
WNA	peptidyl-glutamic acid modification	GO:0018200	0.047	324	PROS1
WNA	negative regulation of osteoblast differentiation	GO:0045668	0.047	324	TNN
WNA	protein alkylation	GO:0008213	0.048	296	ETF1
Molecular Function					
JM	myosin binding	GO:0017022	0.021	495	USH2A
JM	collagen binding	GO:0005518	0.021	425	USH2A
JM	carboxylic acid transmembrane transporter activity	GO:0046943	0.021	328	SLC25A1
WNA	translation factor activity, RNA binding	GO:0008135	0.013	506	EEF1G, ETF1
WNA	sequence-specific mRNA binding	GO:1990825	0.048	1549	ETF1
Cellular Component					
WNA	germ plasm	GO:0060293	0.044	823	TDRD9
WNA	P granule	GO:0043186	0.044	631	TDRD9
SS	Golgi stack	GO:0005795	0.003	1008	GALNT2, GOLGB1

MOLECULAR ECOLOGY

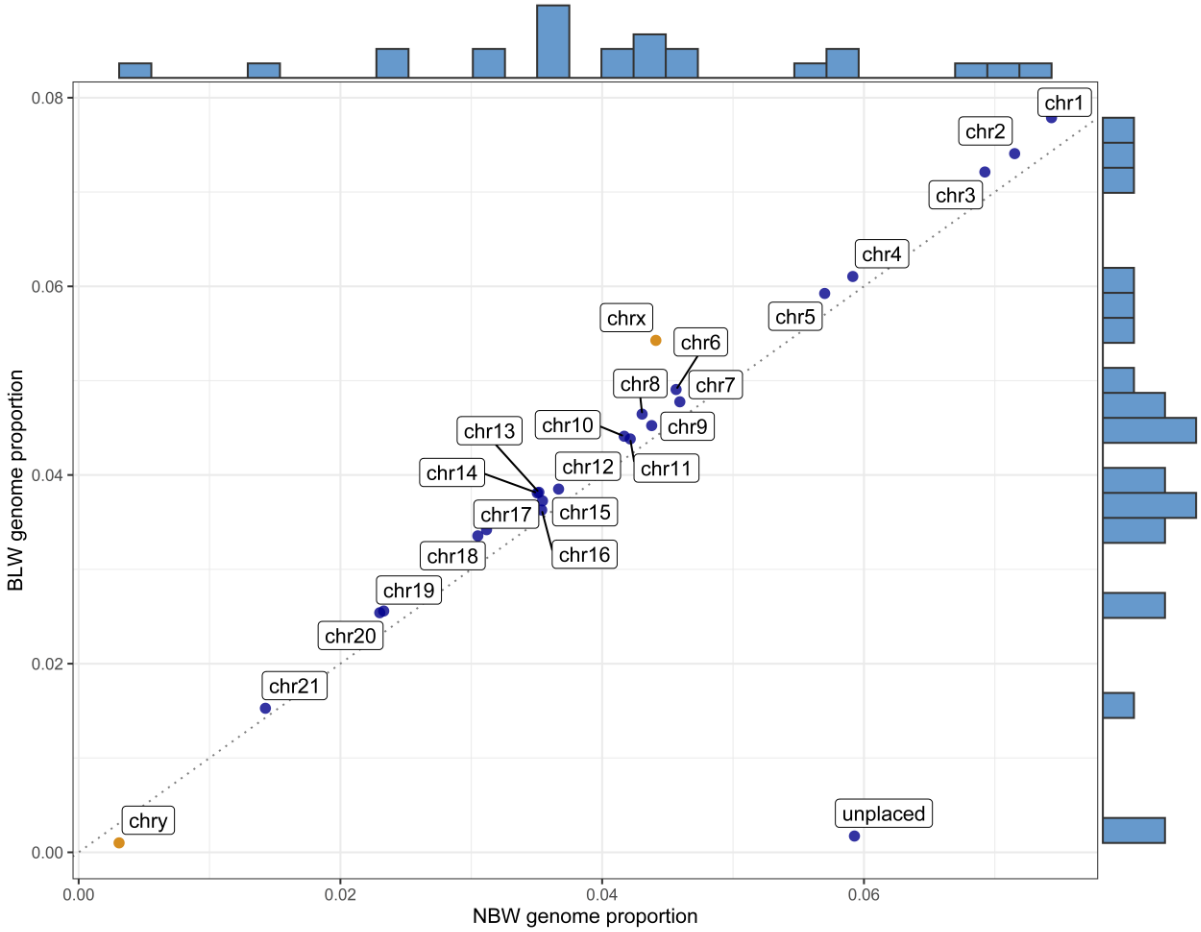

Figure S1. Proportion of autosomes (blue) and sex-chromosome (orange) lengths in final scaffold mapping between the northern bottlenose whale (NBW) and the blue whale (BLW).

MOLECULAR ECOLOGY

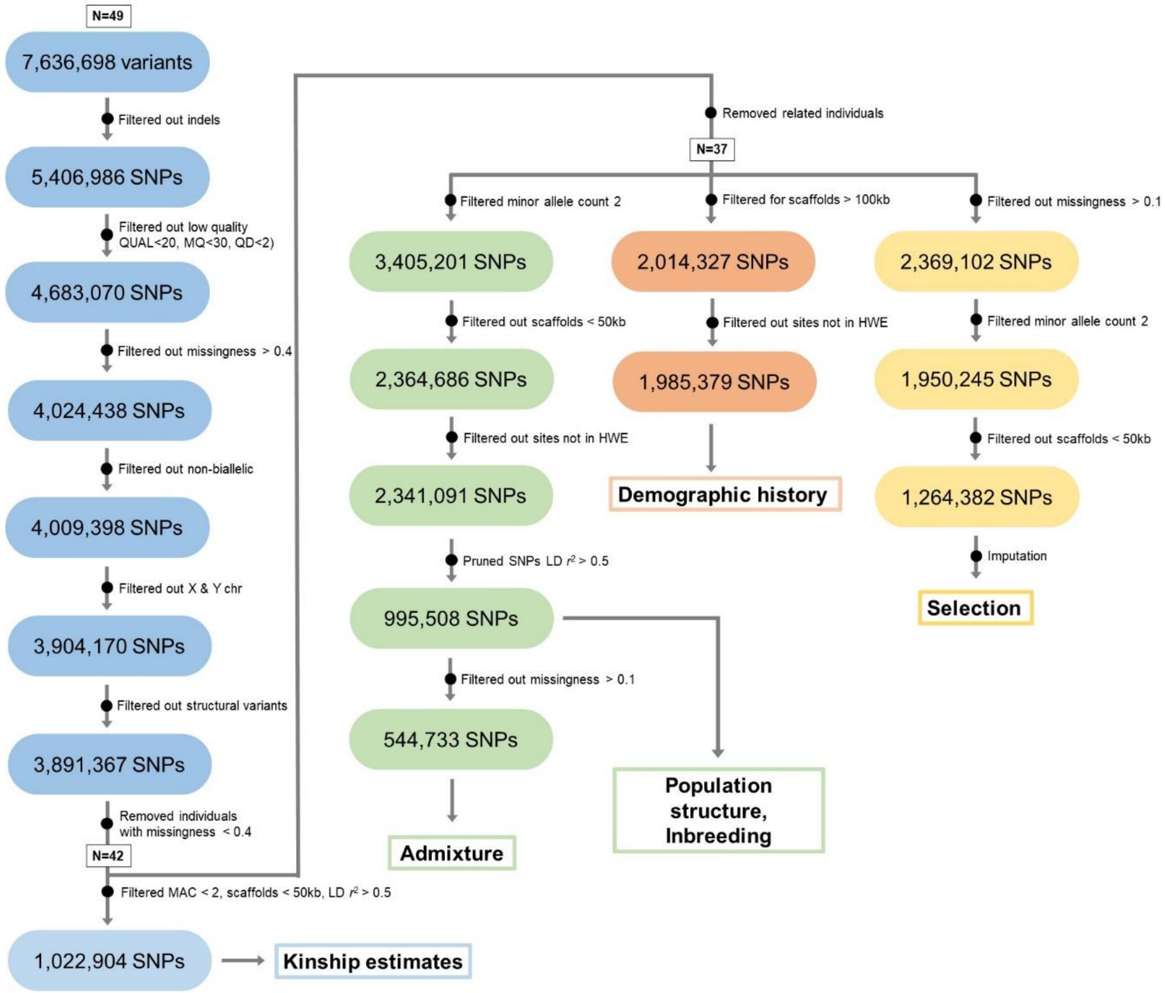

Figure S2. Flowchart of SNP filtering steps in preparation for each northern bottlenose whale analysis.

MOLECULAR ECOLOGY

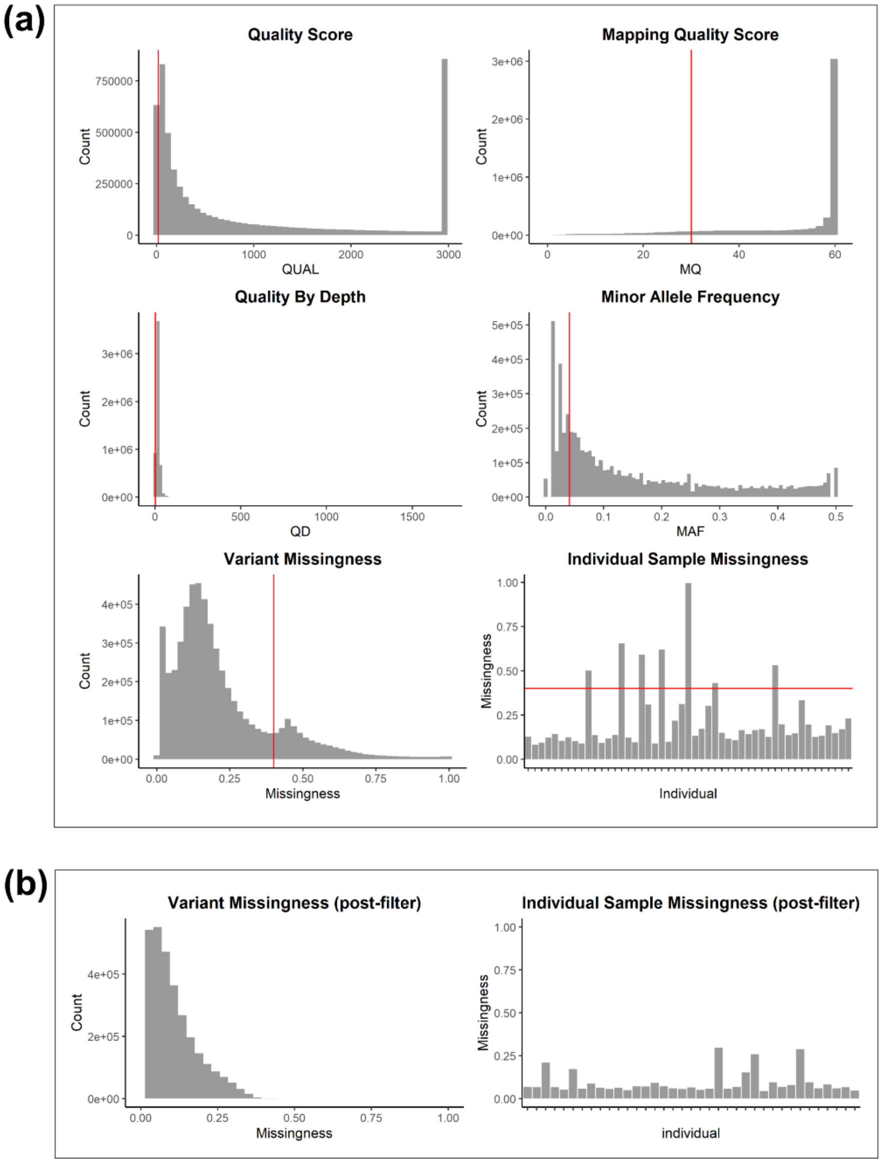

Figure S3. Distributions of northern bottlenose whale SNP metrics (a) prior to filtering for quality, minor allele frequency, and missingness where red lines indicate thresholds used in SNP filtering, and (b) distributions of SNP and individual missingness after filtering.

MOLECULAR ECOLOGY

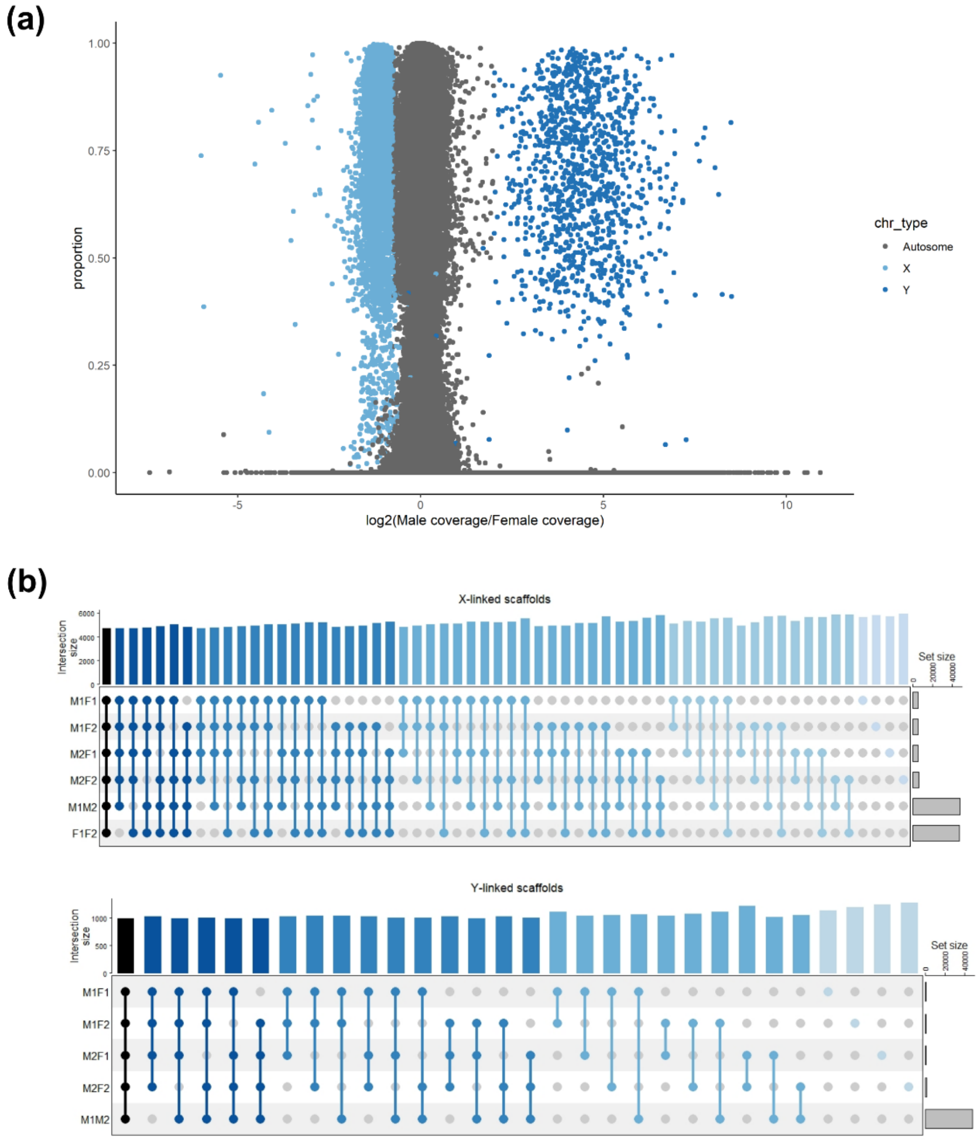

Figure S4. Top Panel (a): Sex-linked scaffold coverage comparison between one male and one female sample, referred to as an "experimental" run to identify the X and Y chromosomes. The y-axis is the proportion of bases in a targeted window by scaffold length and the x-axis reflects an enrichment score, where higher values indicate higher coverage (dark blue cloud) in the male relative to the female consistent with being on the Y chromosome, and lower values indicate approximately half coverage (light blue cloud) in the male relative to the female consistent with being on the X chromosome. Lower Panel (b): Overlap of number of identified X and Y-linked scaffolds throughout combinations between the 6 runs of coverage comparisons using 2 male and 2 female samples demonstrating diminishing returns of adding more comparisons to analyses, with a final number of 4,701 X-linked scaffolds and 984 Y-linked scaffolds.

MOLECULAR ECOLOGY

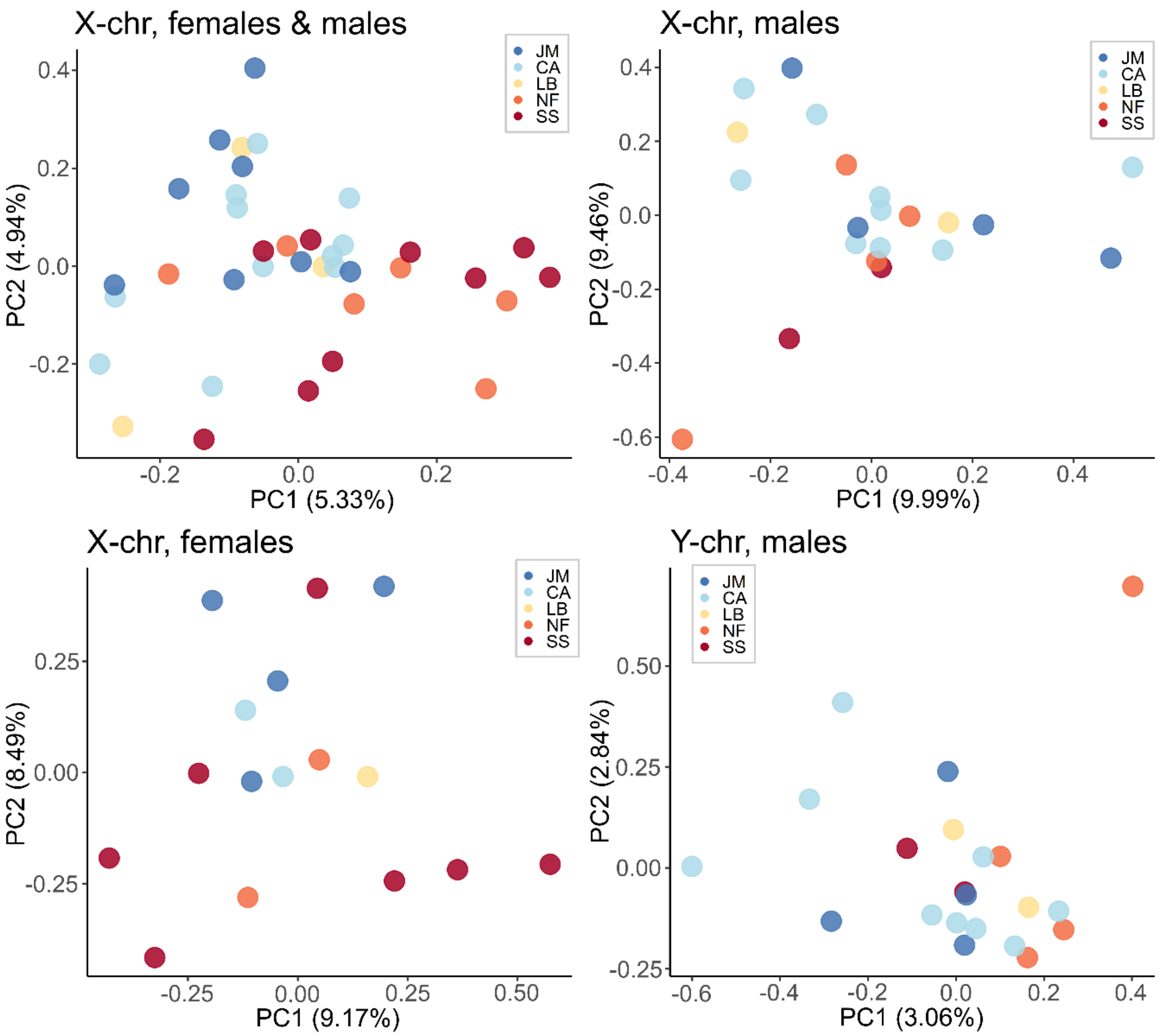

Figure S5. Results from PCAs examining sex-linked SNPs in female and male northern bottlenose whales. JM=Jan Mayen, CA=Canadian Arctic, LB=Labrador, NF=Newfoundland, SS=Scotian Shelf.

MOLECULAR ECOLOGY

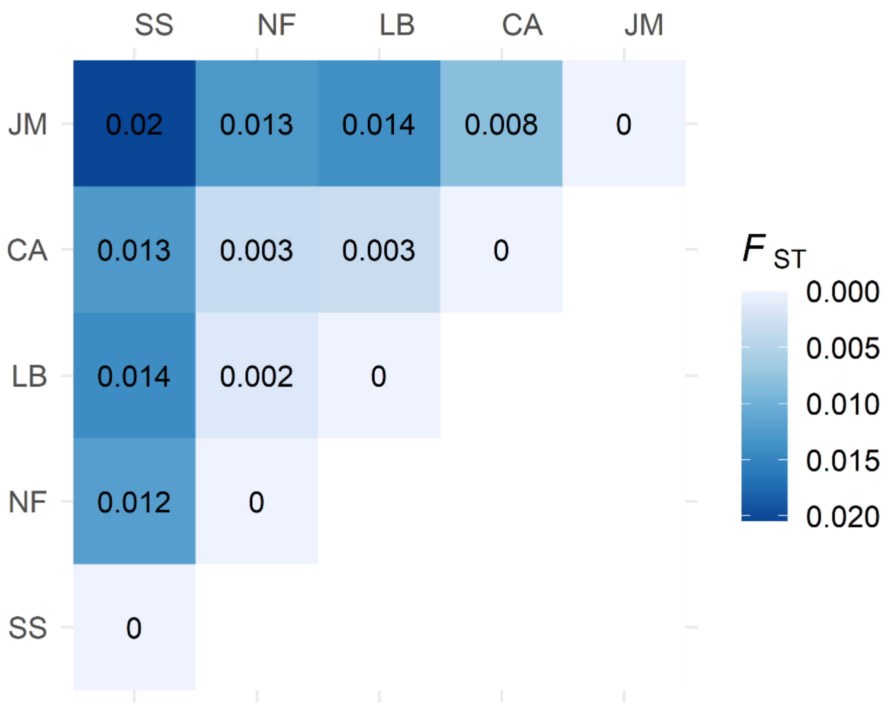

Figure S6. Heatmap of pairwise estimates of differentiation (F_{ST}) in the northern bottlenose whale between sample locations ordered by latitude. JM=Jan Mayen, CA=Canadian Arctic, LB=Labrador, NF=Newfoundland, SS=Scotian Shelf.

MOLECULAR ECOLOGY

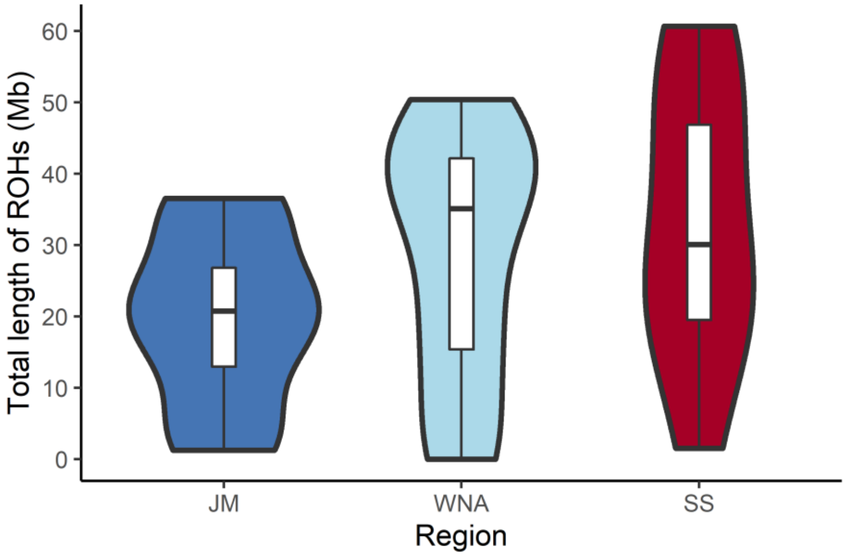

Figure S7. Total lengths of runs of homozygosity (ROH), as an indicator of inbreeding, across three subgroups of northern bottlenose whales. Jan Mayen (JM) displays lowest total length of ROHs, indicating overall lower levels of inbreeding, compared to the western North Atlantic (WNA) and Scotian Shelf (SS) which exhibit wider ranges in lengths of ROH.
